# Ancient DNA confirms diverse origins of early post-Columbian cattle in the Americas

**DOI:** 10.1038/s41598-023-39518-3

**Published:** 2023-08-01

**Authors:** Nicolas Delsol, Brian J. Stucky, Jessica A. Oswald, Charles R. Cobb, Kitty F. Emery, Robert Guralnick

**Affiliations:** 1 Florida Museum of Natural History, University of Florida, Gainesville, FL 32611 USA; 2 Agricultural Research Service, U.S. Department of Agriculture, Beltsville, MD USA; 3 Biology Department, University of Nevada, Reno, Reno, NV 89557 USA; 4 U.S. Fish and Wildlife Service, National Fish and Wildlife Forensic Laboratory, Ashland, OR 97520 USA

**Keywords:** Archaeology, Population genetics, Archaeology

## Abstract

Before the arrival of Europeans, domestic cattle (*Bos taurus*) did not exist in the Americas, and most of our knowledge about how domestic bovines first arrived in the Western Hemisphere is based on historical documents. Sixteenth-century colonial accounts suggest that the first cattle were brought in small numbers from the southern Iberian Peninsula via the Canary archipelago to the Caribbean islands where they were bred locally and imported to other circum-Caribbean regions. Modern American heritage cattle genetics and limited ancient mtDNA data from archaeological colonial cattle suggest a more complex story of mixed ancestries from Europe and Africa. So far little information exists to understand the nature and timing of the arrival of these mixed-ancestry populations. In this study we combine ancient mitochondrial and nuclear DNA from a robust sample of some of the earliest archaeological specimens from Caribbean and Mesoamerican sites to clarify the origins and the dynamics of bovine introduction into the Americas. Our analyses support first arrival of cattle from diverse locales and potentially confirm the early arrival of African-sourced cattle in the Americas, followed by waves of later introductions from various sources over several centuries.

## Introduction

The European colonization of the Western Hemisphere was a crucial event in recent history that arguably shaped the modern world by connecting, through transatlantic travel, cultures and biota of the major landmasses of Afro-Eurasia and the Americas^1,2^. Following humans, it was the arrival of domestic animals from the Eastern Hemisphere that most significantly rearranged the abundance and distribution of species and human activities. Among these, cattle (*Bos taurus*) were particularly impactful as a central part of new post-Columbian (post-1492) economic and social structures^3^, and as a major force in reshaping pre-Columbian landscapes and agrosystems^4,5^. These cultural and environmental impacts of cattle can be attributed in large part to the development of cattle ranching, a management system where animals roam semi-freely on vast tracts of land with little human intervention^6,7^.

The conventional narrative on the introduction of cattle in the post-Columbian Americas based on historical sources suggests that the founding population of the herds in the Spanish colonies was composed of about five hundred animals that were transplanted to the Caribbean islands of Hispaniola, Cuba, and Jamaica^8^. Archival accounts state that these cattle, mostly black Andalusian breeds and piebald *berrenda* cattle, were boarded in the Canary islands and then brought to the Caribbean^9^, and their offspring were then exported to other regions of the Americas by the colonists (Mexico, Panama, Colombia).

Despite the global importance of cattle today^2^, surprisingly little is known about the origin and movement of early post-Columbian cattle in the Western Hemisphere. However, there is extensive genetic research on their early domestication in Europe and archival study of historical documents describing their arrival with the first Spanish colonists^8^, as well as considerable contemporary genetic analysis of modern American heritage breeds such as the Criollo or Creole cattle^10–13^.

The modern heritage cattle of the Americas present a diverse and complex genetic makeup that includes mixed European (T3 haplogroup) and African (T1 haplogroup) ancestries^10,12^. Current descriptions of modern haplotypes are not sufficient to reconstruct how, when, and from where past cattle were imported from and bred in the Americas. To date, genetic analysis of the archaeological remains of colonial American cattle is limited to a single ancient DNA study of partial mitochondrial markers of seven colonial cattle specimens from the site of Sevilla-la-Nueva in Jamaica dated to 1509–1534^14^. Genetic analyses of modern breeds in the Americas revealed combined European and African influences, but left open the question of the chronology and the origins of these introductions to the Americas^10–12^.

### Historical and cultural background

Historical archives document the introduction of the first domestic bovines in the Caribbean by Columbus in 1493. These animals were reportedly boarded on the island of La Gomera in the Canary Islands, a now-Spanish archipelago located 50 miles west of the Moroccan coast of the African continent^15^. However, cattle were not endemic to the Canaries until the 1470 s, when the archipelago was colonized by the Spaniards. Historical archives suggest that the first Canary Island cattle were from the region of Cádiz in southern Spain, brought to the Canaries with the Spanish who colonized the Canary Islands^16^.

According to historical documents, following first cattle brought by Columbus, the founding population in the Americas was a few hundred animals brought to Hispaniola between 1494 and 1512, presumably also via the Canary Island route^8^, although again the exact origin has not been confirmed. This small initial stock is reported to have thrived in the new environments of the greater Caribbean islands and the animals very rapidly multiplied^17^. From this original population, historical documents state that cattle were traded to other Caribbean islands and then by the 1520s to the Gulf coast of Mexico^18–20^ and Panama^8^, on the heels of the intrusion of European settlements into these regions.

Whatever the pathway, cattle were crucial to the emerging Spanish-colonial economy. They were a critical source of the meat, leather, and fats that were central to the European material culture that the Spanish attempted to duplicate in the Americas^21^. Cattle leather also quickly became a major commodity that was traded across the Atlantic Ocean back to the Iberian Peninsula, solidifying the centrality of cattle to the new American colonial economy^21,22^.

### Phylogeographic background of cattle in the Americas

The phylogeographic history of post-Columbian cattle can be described along two main branches, the maternal lineages, defined by mutations in the mitochondrial genome, and the paternal lineages described by divergences in parts of the Y chromosome. Modern cattle emerged out of two separate domestication events. Aurochs (*Bos primigenius*) were first domesticated in southwest Asia around 10,500 years before present, giving rise to the taurine lineages (*Bos taurus*). About 8000 years before present, south Asian wild cattle were then domesticated and gave rise to *Bos indicus*, the zebu or indicine domestic cattle^23^.

Bovine mitochondrial genomes include 10 major *Bos taurus* haplogroups: six T haplogroups (T123, T1, T2, T3, T4, and T5), and the haplogroups P, Q, R, and AA^24,25^. Additionally, two haplogroups correspond to *Bos indicus*, I1 and I2^24^. Within *Bos taurus* the maternal lineages represented by these haplogroups still present a strong geographic structure in the present. The haplogroup T3 is mostly found in European breeds, with about 98.2% of individuals in continental Europe belonging to this group^11^. On the other hand, haplogroup T1 is particularly prevalent in African breeds (91.3% of individuals). Some of the south European breeds, particularly Iberian cattle, show a higher degree of African influence (9.4% of individuals), with some specimens belonging to the T1 haplogroup, potentially due to millennia of cattle interactions between North Africa and Europe across the Strait of Gibraltar^11,26–29^.

In the Americas, the mitogenomes of the modern Creole breeds, the heritage bovine varieties deemed to descend from colonial cattle, present a complex genetic makeup composed mostly of European ancestry with a notable influence of African varieties^11,30–33^. More specifically, modern Creole breeds show a significant contribution of the T1a, a matriline that is mostly found in Africa but also in significant proportions in southern European breeds. These American heritage cattle also reveal an “African-derived American” sub-haplogroup of T1 (AA-T1c1a1) identified in only a few groups (in Baja California and Chihuahua in Mexico, Caracú in Brazil, and Pampa Chaqueño in Paraguay)^10,14,30^. These findings are suggestive of early African cattle being present on American continents, but the exact timing of their arrival is unknown. This is further supported by Speller and colleagues’ study of archaeological cattle specimens from sixteenth century Jamaica. Their ancient mtDNA study examined 1000 bp sequences covering parts of the mitogenome D-loop and revealed that six out of seven specimens were closely related to the T3 haplogroup of primarily European breeds and one to the T1 haplogroup of African/southern European breeds^14^. However, their results did not provide further insight into the arrival of African cattle to the Americas.

**Figure 1:**
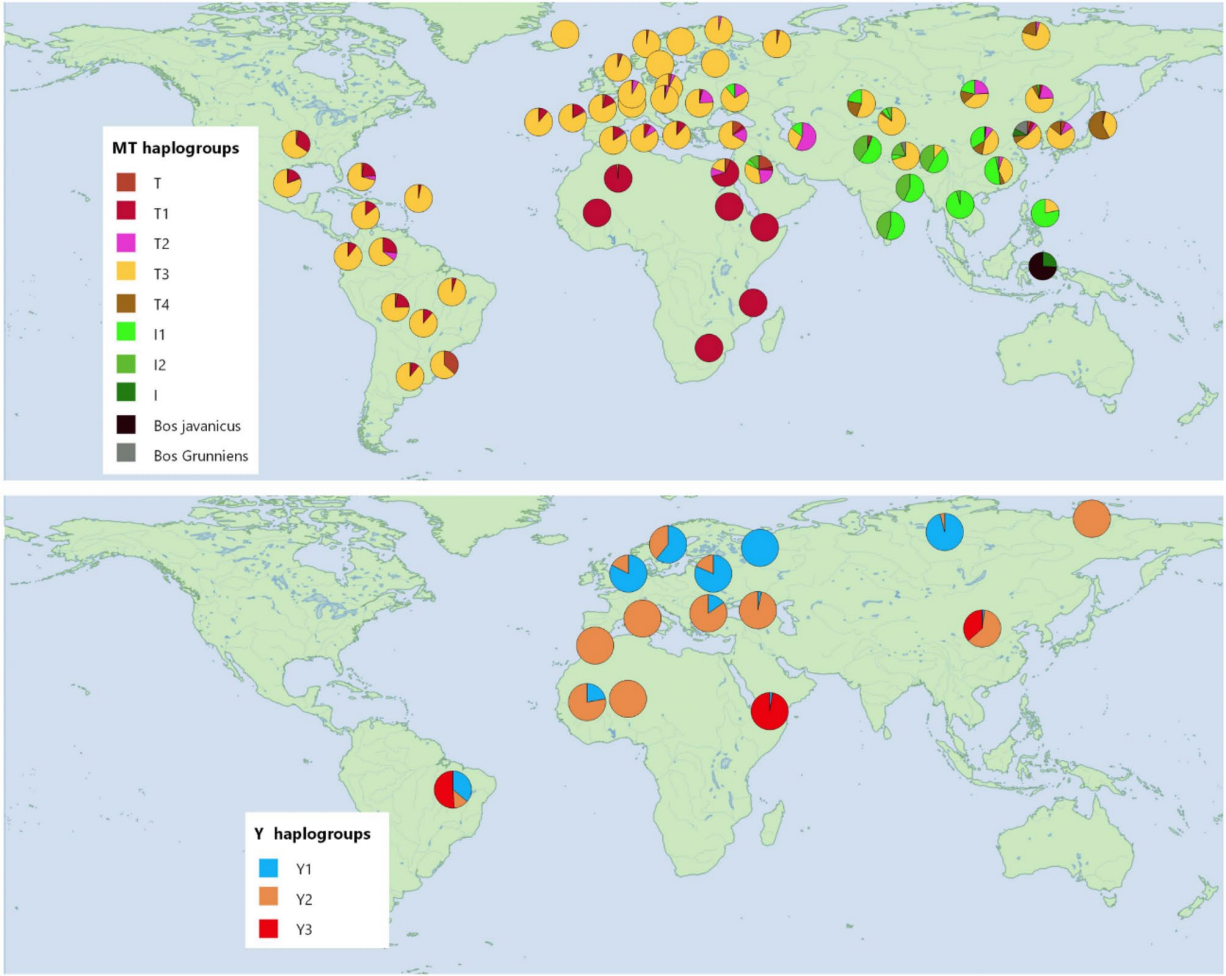
World distribution of cattle mitochondrial and Y haplogroups. Above: distribution of cattle maternal lineages (data from Lenstra et al.^35^). T combines the haplogroups T, T1’2’3’ and T5, Bali cattle (*Bos javanicus*), yak (*Bos grunniens*) ratios are included in some east Asian locations. Haplogroups P, Q, and R do not figure on the map as they are very rare clades found in Eurasia. Below: distribution of cattle paternal lineages (data from Di Lorenzo et al.^36^).

The paternal lineages based on modern genetic data have proven more complex. Prior studies examined both archaeological and modern specimens from European, African, and Asian breeds^34^. The data generated in this Y chromosome study was based on single nucleotide polymorphisms (SNPs)^18^ and identified three main Y haplotypes through mutations (five SNPs and one insertion-deletion) and one microsatellite in five Y chromosome introns (DDX3Y intron 1, DDX3Y intron 7, UTY intron 19, ZFY intron 4, ZFY intron 5). The authors found that haplotype Y3 is associated with indicine lineages (*Bos indicus*), and two haplogroups Y1 and Y2 present a north–south gradient in Western Europe, with Y1 more common in north European populations and Y2 found in higher frequencies in the South. In the Americas, the modern heritage Creole breed patrilines show evidence of moderate indicine (Y3; zebu; South Asia) introgressions (39.5% of Y haplogroups)^11^ (Fig. 1).

The current study aims to supplement the historical documentary and modern and mitogenomic aDNA evidence with an analysis of complete archaeological mitogenomes from historical cattle. It also constitutes the first attempt in this region at extracting Y chromosome data from archaeological cattle. Our main goal is to resolve areas of uncertainty among the proposed pathways that led to the distribution of historical and modern cow breeds in the Americas. Here we sample ancient DNA from archaeological specimens from a regional expanse that includes Hispaniola in the Caribbean to central Mexico in Mesoamerica, and a chronological span from the early 16^th^ to the late eighteenth centuries, with most specimens dating to the early periods (12 from sixteenth century specimens). This early period is critical to our understanding of the dynamics of the introduction of cattle in the region because the colonial ranching industry was implemented in many regions of Spanish America during this time. These data are used to answer a set of outstanding questions:

What is the potential geographic origin of the founding population(s) of post-Columbian cattle? Given that modern heritage cattle in the Americas have a strong imprint of African breeds, what is the timeline for this introgression across these breeds? Is this genetic influence on modern American heritage breeds the result of earlier exchanges of cattle between North Africa and southern Spain prior to Caribbean migration^26,37^? Or is it evidence of potential direct introductions of cattle from Africa to the Americas^10,12^? If confirmed, the latter scenario could suggest very early introductions of African cattle with the first Spanish colonists to the Americas, or it may be related to the increasing exchanges between West Africa and the Americas at the end of the sixteenth century associated with the slave trade.

A second pressing question is what is the history of early expansion of cattle across the circum-Caribbean after their first documented arrival with Columbus on his arrival in Hispanola^15^? Historical literature suggests a single founding population in Hispaniola spreading through trade across the Caribbean and then to the mainland. Does the high genetic diversity recorded among American heritage cattle at present thus reflect later colonial (late 1500s onward) and post-colonial introductions, or is it possible that even the earliest cattle were much more diverse than currently envisioned?

## Results

**Figure 2:**
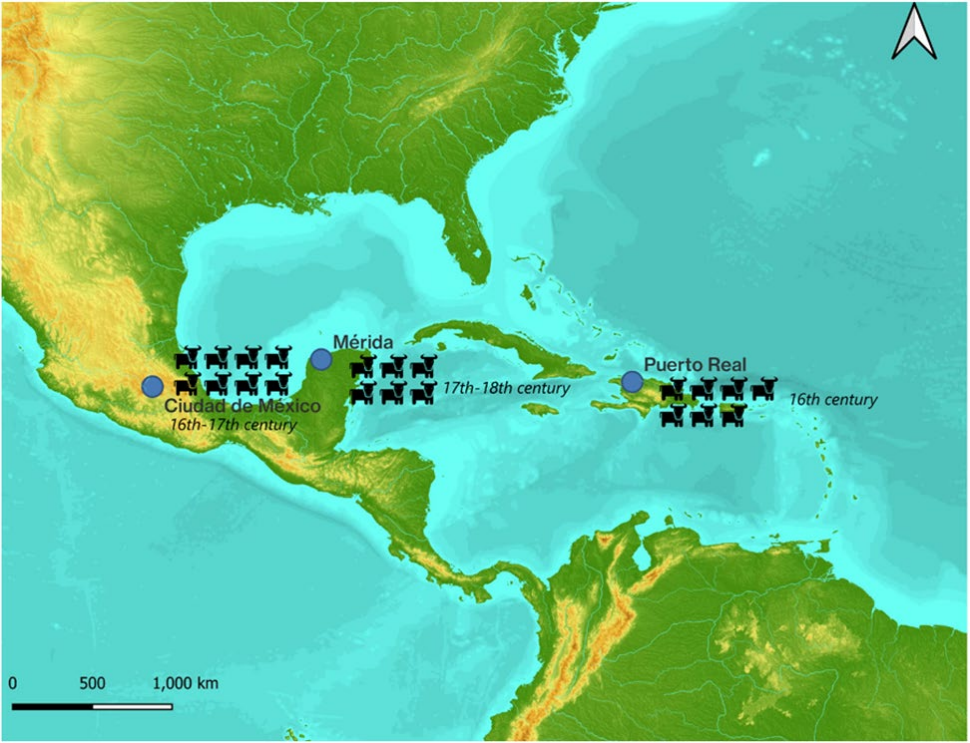
Map of the Circum-Caribbean region with the location of the three sites (original map by N. Delsol, cartographic data from USGS EROS http://eros.usgs.gov/, source: NASA/METI/AIST/Japan Spacesystems and U.S./Japan ASTER Science Team).

We sequenced the mitogenomes from 21 archaeological specimens (Table 1, Fig. 2) and for eight of these, we also attempted to sequence the Y chromosome introns. Out of these eight specimens, three provided usable data and could thus be included in the analysis.

**Table 1:** List of samples analyzed in this study.

Name	Country	Chronology	Genbank Acc#	# raw reads	# on-target	Sample
Puerto Real 2	Haiti	Sixteenth century	OP858992	15,957,102	674,107	Petrous bone
Puerto Real 4	Haiti	Sixteenth century	OP858993	25,191,494	3,675,646	Petrous bone
Puerto Real 5	Haiti	Sixteenth century	OP858994	21,918,918	13,991	Petrous bone
Puerto Real 6	Haiti	Sixteenth century	OP858995	11,599,942	779,641	Molar
Puerto Real 7	Haiti	Sixteenth century	OP858996	13,143,584	464,713	Molar
Puerto Real 8	Haiti	Sixteenth century	OP858997	10,212,936	4,833	Molar
Puerto Real 9	Haiti	Sixteenth century	OP858998	16,325,358	71,265	Petrous bone
Merida 11	Mexico	Seventeenth–eighteenth century	OP858999	13,724,392	1,334,056	Molar
Merida 12	Mexico	Seventeenth–eighteenth century	OP859000	13,373,196	1,364,911	Molar
Merida 13	Mexico	Seventeenth–eighteenth century	OP859001	25,945,016	58,108	Premolar
Merida 14	Mexico	Seventeenth–eighteenth century	OP859002	12,185,490	2,308,409	Metacarpal
Merida 15	Mexico	Seventeenth–eighteenth century	OP859003	39,209,512	4,853,989	Petrous bone
Merida 16	Mexico	Seventeenth–eighteenth century	OP859004	17,786,940	2,303,864	Molar
CDMX Xochimilco 17	Mexico	Sixteenth–seventeenth century	OP859005	30,907,292	13,652,003	Carpal (scaphoid)
CDMX Xochimilco 18	Mexico	Sixteenth–seventeenth century	OP859006	39,080,792	10,435,819	Humerus
CDMX Justo Sierra 19	Mexico	Sixteenth–seventeenth century	OP859007	1,759,934	209,772	Carpal (scaphoid)
CDMX Justo Sierra 20	Mexico	Sixteenth–seventeenth century	OP859008	17,461,756	8,538,852	Carpal (semilunar)
CDMX Justo Sierra 21	Mexico	Sixteenth–seventeenth century	OP859009	17,369,222	9,440,148	Carpal (semilunar)
CDMX Bellas Artes 22	Mexico	Sixteenth–seventeenth century	OP859010	40,683,574	19,444,947	Carpal (scaphoid)
CDMX Bellas Artes 23	Mexico	Sixteenth–seventeenth century	OP859011	13,453,292	2,892,260	Humerus
CDMX Bellas Artes 24	Mexico	Sixteenth–seventeenth century	OP859012	17,221,538	5,927,346	Molar

Seven specimens with genetic data come from the 16th-century Spanish town of Puerto Real, located on the northeastern coast of Haiti. Puerto Real was a major center of Spanish colonialism in the region from its foundation in 1503 until its abandonment at the end of the sixteenth century^38^. The town’s economic life revolved principally around cattle ranching and the trade of hides and other cattle byproducts. Six specimens are associated with a Franciscan convent from the site of La Ciudadela in Merida, Yucatan (Mexico)^39^. This city, which was founded in 1542 by Francisco de Montejo, a lieutenant of Hernán Cortés, at the location of the Postclassic Maya city of Tíhoo, was an important urban center, home to Spanish residents, Maya natives, and workers of African descent. The archaeological context where the sample comes from dates from the late seventeenth–eighteenth century. Eight specimens come from three domestic deposits in Mexico City: Xochimilco (2 specimens, sixteenth century), Justo Sierra (3 specimens, sixteenth century), and Bellas Artes (3 specimens, seventeenth century). Xochimilco was a Contact-era indigenous settlement located south of Mexico City^40^ while Justo Sierra, a Spanish elite early colonial house^41^, and Bellas Artes, a Franciscan convent^42^, are centrally located in the modern city limits.

### Haplotype network of the maternal lineages

**Figure 3:**
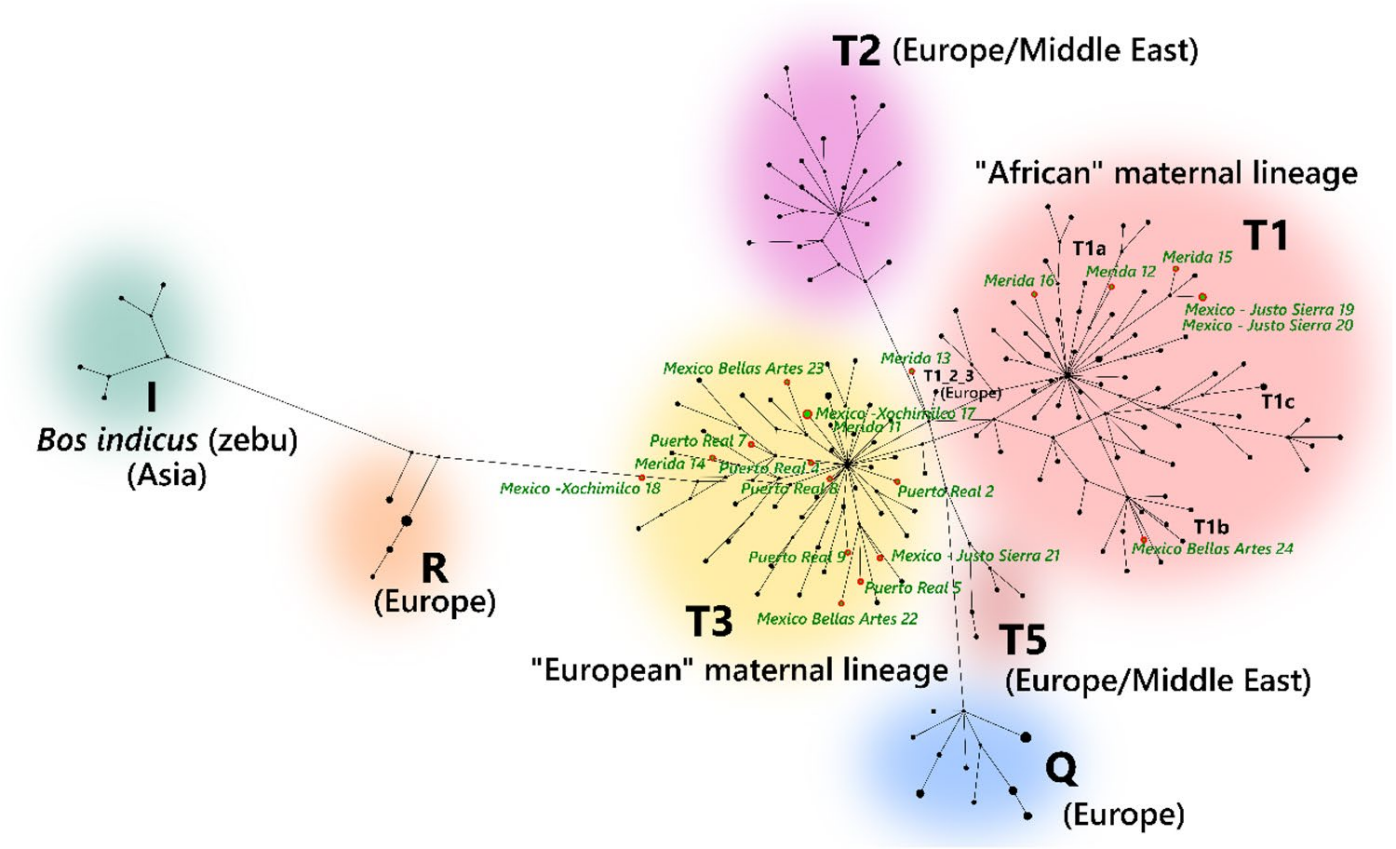
Median joining network of *B. taurus* and *B. indicus* mitogenomes with 21 archaeological genomes (archaeogenomes labeled in green).

The phylogenetic structure of the maternal lineages was visualized using a median-joining network, an approach based on Kruskal’s algorithm for generating minimum spanning trees and Farris’s maximum-parsimony (MP) heuristic algorithm^43,44^. For comparison purposes, 159 modern reference sequences were selected from among published complete cattle mitogenomes analyzing the distribution of primarily T1 and T3 maternal lineages found mostly in Europe, Africa, and the Americas^10,12,24,25^.The resulting median-joining network places the 21 archaeological cattle mitogenomes in relation to the 159 published modern mitochondrial sequences of known haplotype. Mitochondrial genome data from the archaeological specimens clustered within the *Bos taurus* lineages and are mostly distributed between two major taurine haplogroups: haplogroup T1 (“African”) and haplogroup T3 (“European”) (Fig. 3). Fourteen specimens, including all the specimens from the early 16th-century contexts of Puerto Real and Xochimilco, one specimen from Justo Sierra (16th c.), two specimens from Bellas Artes (17th c.), and two from Merida (17th c.) belong to the T3 haplogroup. Six other specimens of mid-sixteenth to eighteenth century contexts cluster in the T1 haplogroup. These include two from Justo Sierra, one from Bellas Artes, and 4 from the later Merida deposits.

Most of these T1 specimens present more affinities with members of the T1a subhaplogroup which is equally found in Africa and Southern Europe. However, one individual from Mexico City (Bellas Arte 24) is connected to the T1b matriline and another one from Merida (Merida 16) falls into the T1d subclade. Both of these taurine subhaplogroups are almost exclusively found today on the African continent^25^. One specimen from Merida (Merida 13) seems to hold an intermediate position, clustering more closely with the small T123 ancestral haplogroup identified in some Italian breeds^24,45^. All specimens from the earliest assemblages, such as Puerto Real and Xochimilco, both dating from the early sixteenth century, belong to the T3 haplogroup. Later assemblages from the sites of Bellas Artes, Justo Sierra, and more particularly Merida, present more diversity in terms of haplogroups, with a significant T1 component.

### Maternal lineages maximum-likelihood tree

We used RAxML to obtain a phylogeny of the 21 archaeological and 42 complete modern taurine mitogenomes with indicine cattle sequences as outgroup (GenBank # NC005971 and EU177869)^46^ (Fig. S1). This approach uses a maximum likelihood approach to estimate the best tree topology and branch lengths based on input sequence data.

The resulting phylogenetic tree reveals patterns already observed in the haplotype network, namely that the post-Columbian cattle population can mostly be divided into two main groups that correspond to the two major taurine haplogroups found in the Eastern Hemisphere: six individuals in the T1 haplogroup and 14 in the T3. The T1 and T3 haplogroup clade has 100% bootstrap support. Within this larger clade, robust support for finer scale population-level associations is weaker. Five out of the six archaeological specimens of the T1 haplogroup present more affinities with individuals of the T1a subhaplogroup (57% bootstrap support), a clade that is commonly found in Africa but also in some southern European breeds^25^. The Bellas Artes 24 specimen falls into the T1b1 subhaplogroup and relates more closely to African breeds such as Domiaty or Sheko cattle (52% bootstrap support). This attribution to T1b1 is confirmed by specific SNPs identified in the Bellas Artes 24 sequence that characterize this subclade, namely two transitions at positions 7,542 and 16,022 of the Bovine Reference Sequence^25,47^.

Among the archaeological samples that belong to the T3 haplogroup, all are closely related to Southern European breeds, which is consistent with an Iberian origin of the animals. Some of these archaeological individuals also group into distinct subclades: for example, the clade including Xochimilco 17 (Mexico City), Merida 11, and Puerto Real 6, or the subgroup including Puerto Real 5, Puerto Real 8, Puerto Real 7, Bellas Artes 22 (Mexico City), and Justo Sierra 21 (Mexico City). Finally, our ML phylogeny confirms the outlier status of the Merida 13 specimen. Interestingly, no archaeological mitogenome of post-Columbian cattle present a strong affinity with modern Creole varieties of the T1c subhaplogroup.

### Paternal lineages

Out of eight archaeological specimens sampled for Y chromosome markers analysis, only three provided exploitable results: Justo Sierra 20, Bellas Artes 22, and Merida 15. The sequences of these three specimens almost completely covered the five Y chromosome introns used to define the main male bovine lineages (DDX3Y intron 1, DDX3Y intron 7, UTY intron 19, ZFY intron 9, and ZFY intron 10). Together, these five regions of the Y chromosome represent a total of 3169 bp. Three main male lineages are defined using five SNPs, one insertion-deletion, and one microsatellite: Y1, Y2, and Y3 (Table 2)^34,48^. The alignment of the three archaeological sequences with modern comparative specimens revealed that they all belong to the Y2 haplogroup. Geographically, this haplogroup is currently found in many cattle populations, but it is mostly prevalent in southern European cattle and to a lesser extent African populations (Table 3). Interestingly, the modern Creole breeds, deemed to descend from cattle introduced by the Spaniards during the colonial period, present a lesser frequency of this male lineage^11^. While this could suggest a diverse origin of colonial male lineages, the limited Y chromosome dataset may also be indicative of a modern turnover in male ancestries as indicine bulls were imported later to improve breeds^49^.

**Table 2:** Main Y haplogroups genetic markers used in this study (from^34^).

Y haplogroup	Taxon	DDX3Y_1	DDX3Y_1 microstatellite	DDX3Y_7	UTY_19	ZFY_9	ZFY_10	Indel ZFY_10
Y1	*Bos taurus*	C	(AT) × 10	C	C	C	C	–
Y2*	*Bos taurus*	C	(AT) × 10	C	A	C	C	GT
Y3	*Bos indicus*	T	(AT) × 8	T	A	T	T	GT

*Merida 15, Justo Sierra 20, Bellas Artes 22.

DBX3Y intron 1 SNP, position 425 in in GenBank AY928816; DDX3Y intron 1 microsatellite, position 363 in AY928816; DDX3Y intron 7 position 165 in AY928817; UTY19, position 423 in AY936543; ZFY4, position 120 in AY928828; ZFY5 SNP, position 609 in AY928827; ZFY5 indel, position 651 in AY936548.

**Table 3:** Y haplogroups frequency in different modern cattle populations (from Ginja et al*.*^11^).

Y Haplogroup frequency	Creole	Iberian	British	Continental	African	Indicine	Total modern
Y1	0.35	0.292	0.857	0.264	0.088	0	0.332
Y2	0.254	0.708	0.143	0.736	0.424	0	0.465
Y3	0.396	0	0	0	0.488	1	0.203

### Genetic diversity

**Figure 4:**
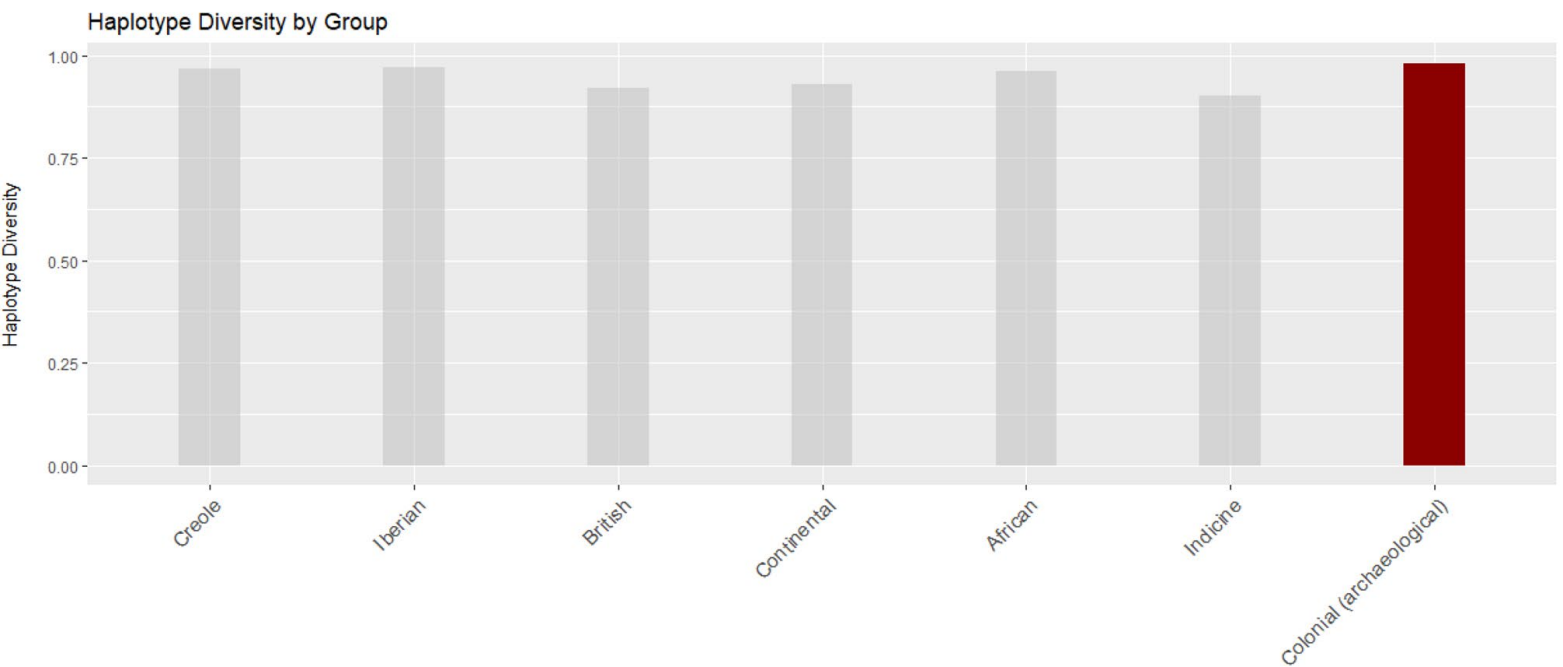
Bar chart of the haplotype diversity indices (H) in different modern populations versus archaeological post-Columbian cattle (modern data Ginja et al*.*^11^).

The variation and diversity of colonial maternal lineages was estimated through the measurement of haplotype diversity in the D-loop portion (700 bp) of the archaeological sequences (Fig. 4, Table 4) using the “Polymorphism Data” tool of DNAsp v.6.12.03^50^. Focusing solely on this non-coding region of the archaeological sequences allowed for broader comparisons with published results on large modern datasets of cattle worldwide^26^. Despite its small size, the overall genetic diversity of the archaeological colonial cattle is particularly high (H = 0.981), even when compared with highly diverse populations such as the Iberian (H = 0.972) or Creole (H = 0.966).

**Table 4:** Mitochondrial diversity indices and mt haplogroup frequencies for modern cattle populations in comparison with archaeological specimens (from Ginja et al*.*^11^).

	Creole	Iberian	British	Continental	African	Indicine	Total modern	Colonial (archaeological)
MT								
# breeds	33	36	6	4	9	5	93	N/A
# specimens	460	627	101	55	161	66	1470	21
Haplotype diversity (H)	0.966	0.972	0.92	0.931	0.961	0.903	0.942	0.981
# haplotypes	117	248	52	31	78	27	463	18
MT Haplogroup frequency								
T	0	0	0.01	0	0.012	0	0.002	0.05
T2	0.009	0.021	0	0.018	0.025	0	0.015	0
T3	0.713	0.868	0.99	0.982	0.05	0.5	0.726	0.67
Q	0.03	0.01	0	0	0	0	0.014	0
T1	0.165	0.093	0	0	0.832	0.121	0.188	0.29
T1c1a1	0.083	0.01	0	0	0.081	0.364	0.055	0
I	0	0	0	0	0	0.015	0.001	0

Comparing the haplogroup distribution of the colonial cattle with modern populations strengthens this suggestion of relatively high diversity of the historical stock. In the archaeological samples introduced here, the European T3 haplogroup is prevalent (66.6%), but in lesser proportions than in the Continental European (98.2%), British (99%), and even the Iberian and Creole breeds (86.8% and 71.3%, respectively). The T1 haplogroup, overwhelmingly present in southern European African breeds (83.2%), is also found among the historical specimens (29%).

## Discussion

The aims of this study were twofold: (1) to identify the possible geographic origins of early colonial cattle in regions that were colonized by Spanish and other European settlers, and (2) to investigate the dynamics of this introduction, with a particular focus on the diversity of the early colonial cattle. Our analyses of the maternal and paternal lineages of colonial cattle provide contrasting results between the origins of female versus male cattle to the Americas that echo results from earlier studies on modern American breeds. Our results, although based on partial genomic data, provide critical details to reconstruct the genetic history of cattle in the Americas and refine our understanding of the timeline of their introduction.

**Figure 5:**
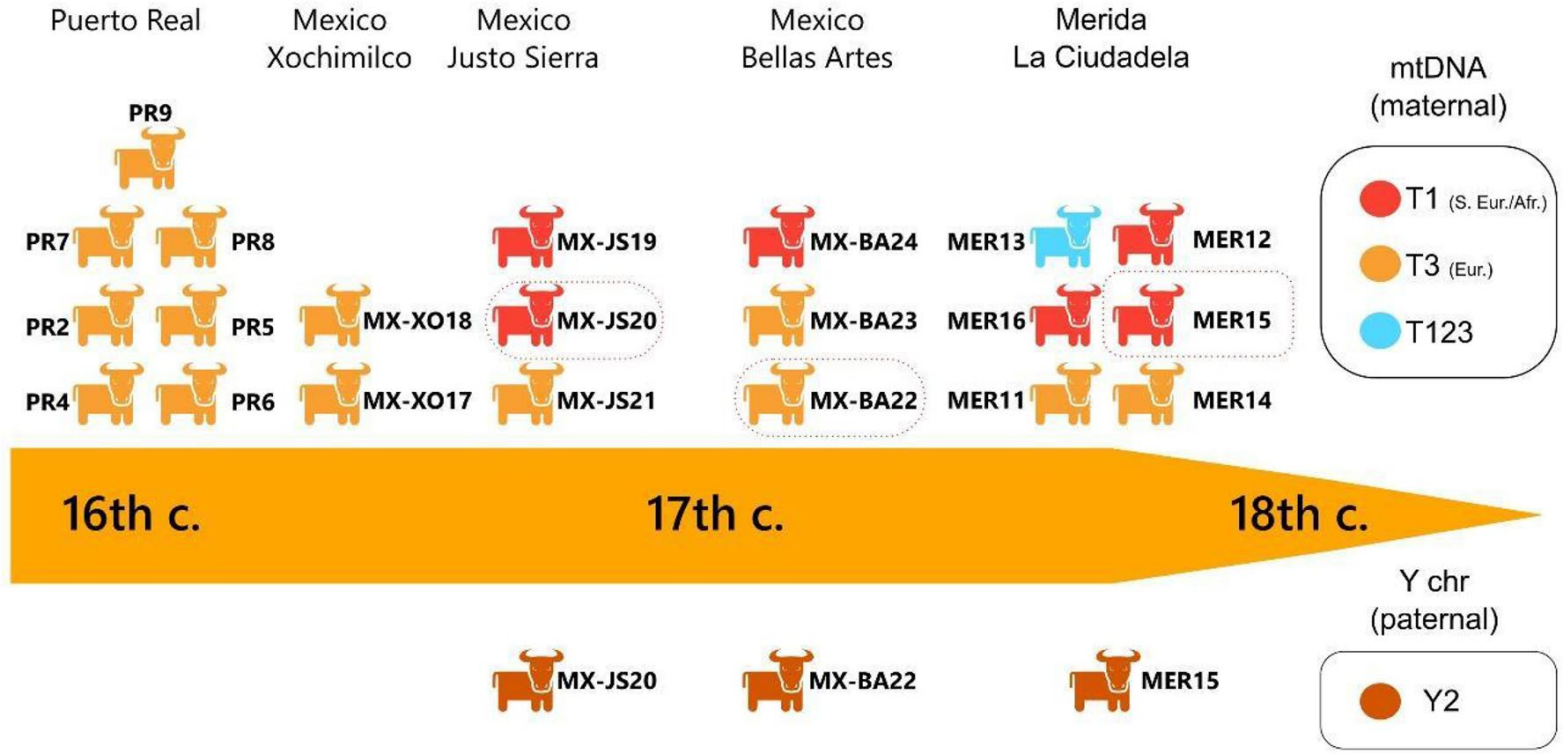
Synthesis diagram showing the genetic makeup of post-Columbian cattle and their chronological evolution.

Concerning the geographic origins of colonial cattle, the mitochondrial evidence consistently suggests that the first cattle in the Western Hemisphere are from haplotypes today found in southern Europe, Africa (T1) and broadly Europe (T3). The Merida 13 sample from the seventeenth–eighteenth century deposit of Merida is in an intermediate position, likely belonging to the T123 taurine haplogroup, today found in southern Europe. Placing these archaeological sequences in chronological order (Fig. 5), the nine earliest specimens dating from the early to mid-sixteenth century (Hispaniola: Puerto Real, Mexico: Xochimilco) all belong to the T3 haplogroup. Later archaeological contexts (Mexico: Bellas Artes, Justo Sierra, and Merida) present a greater haplogroup diversity with individuals belonging to the T1, T3 and T123 haplogroups. In addition to the haplotype diversity, mitochondrial data suggests that the introduction of bovines in the Western Hemisphere was more complex than initially inferred from the historical documentation.

The archaeological male lineages identified in the archaeological specimens all relate to the Y2 haplogroup, mostly found in the southern European breeds, but also in significant proportions in Africa. These data are consistent with the probable origins of these bulls in Iberia, or potentially in Africa. Two of the bulls also belong to the T1a maternal lineage (Mexico Justo Sierra 20 and Merida 15) while one is related to the European T3 clade. Considering that the colonial breeding practices likely relied on only a small subset of males used for breeding, there must have been strong human selection for bull traits. Our dataset on colonial paternal lineages is very limited, but they provide important considerations for further studies on early colonial cattle.

The establishment of European driven economic practices in the Western Hemisphere may have led to increased genetic diversity of cattle breeds to be imported to the region through time. While it is difficult to assess exact breed origins of the colonial cattle based on the different haplogroups, the ancient maternal lineages provided herein reveal a different narrative that includes increasingly diverse sources for later cattle, supported by our evidence for increasing mitochondrial haplogroup variety over time. As well, in the later archaeological contexts (Merida, and Bellas Artes and Justo Sierra in Mexico City), there is an increasing presence of bovines associated with the T1 haplogroup, the “African” bovine clade (found in southern Europe and Africa). The high diversity of colonial cattle, in a population that had been only recently introduced in the Americas, in addition to the distribution of haplogroups could be support for a hypothesis of a variety of origins of this early stock. However, such relatively high genetic variability could also be related to population bottlenecks in modern breed formation.

### African origins?

The direct introduction of African cattle to the Americas has been an ongoing question raised in earlier studies based on modern^10,12^ and archaeological genetic material^14^. Recent analyses on a large dataset of mitochondrial, Y chromosome and autosomal markers confirmed the direct influence of African cattle on Creole breeds at an unknown point in time^11^.

The analysis of the 21 colonial mitogenomes herein links five of these six T1 archaeological individuals with the T1a subhaplogroup, most prevalent among African breeds (although also found in modern south European breeds such as the Italian Marchigiana or the French Limousine)^25^. Given the sustained relations between North Africa and Spain over time, we cannot discard the possibility that this presence of individuals belonging to the T1a subhaplogroup is a result of ancient African influence in the Iberian stock. On the other hand, our analyses reveal that the Bellas Artes 24 (Mexico City) specimen belongs to the T1b subhaplogroup. This group is particularly rare in Europe and has no occurrences in living heritage breeds in the Americas^25^. There fore, our results are highly suggestive of an African origin for this individual, dated to the early seventeenth century.

These affinities between the Bellas Artes 24 specimen mitochondrial sequence and a typical African cattle haplogroup such as T1b, lends credence to the idea that some cattle specimens could have been introduced directly from Africa as early as the first half of the seventeenth century in regions such as central Mexico. This hypothesis, which is strengthened based on results here, could be confirmed through further analyses focused on the nuclear genome that would more precisely detail the ancestries and geographic origins of historical cattle.

The question of the potential African origin of some colonial cattle is of immense historical significance and has deep social and cultural ramifications, particularly when considering the central role played by African workers in setting up the ranching industry in the colonial Americas. Our archaeological genetic evidence of cattle parallels these documented aspects of the early Spanish Empire in the Americas: the organization of the colonial labor system, the timing of the African slave trade, and the high specialization of enslaved workers in cattle management. While the European colonists held most of the economic and political power, they relied on a diverse workforce, mainly composed of Native and African coerced workers to generate their wealth in both urban and rural regions from Mexico to Peru^51–53^. In rural areas, the knowledge of these laborers and their adaptability to the tropical conditions of some parts of Spanish America were also particularly valued^54^. Workers of African descent were particularly prominent in one craft in particular: cattle ranching. Numerous historical sources suggest that this enslaved workforce played a crucial role in the management of the numerous herds of cattle that roamed semi-freely in different regions of the Americas (Caribbean, Gulf Coast of Mexico, Oaxaca, South American Llanos)^55,56^. Overall, it seems that complex, trans-colonial collaborations opened a potential conduit for a wide array of products (including cattle) alongside enslaved Africans into the Caribbean and Mesoamerican regions after the 1550s CE.

Our data, while not fully conclusive, further support the hypothesis that cattle were also imported from Africa to the Americas, highlighting the central role of African herders in the emergence of the new agricultural landscape mainly based on cattle ranching. The chronology of these introductions (late sixteenth–early seventeenth century) is also consistent with the rise of the transatlantic slave trade that occurred in the second half of the sixteenth century^57^. These colonial entanglements played a central role in the ongoing genetic diversification of cattle in Spanish America.

## Materials and methods

### Archaeological specimens

A total of 23 bone samples were selected for the DNA analysis. The specimens come from the sites of Puerto Real in Haiti (10 samples), La Ciudadela–Merida in Yucatan, Mexico (6 samples), and Mexico City (8 samples)(Table 1). Of these, two samples (Puerto Real 1 and Puerto Real 10) did not return any genetic data.

The specimens from Puerto Real and Merida are currently curated in the Environmental Archaeology Program (EAP) of the FLMNH with permission of the Haitian and Mexican governments. Permission for their use for destructive analysis research was provided by the curators of the Historical Archaeology (Cobb) and Environmental Archaeology (Emery) programs, under EAP Research and Publication Permit # R0121). Sampling was conducted with advice from Kitty Emery (Curator) and Nicole Cannarozzi (Collection Manager) following the EAP protocols for ethical destructive analysis. The samples from Mexico City, initially curated at the Laboratorio de Arqueozoología of the Instituto Nacional de Antropología e Historia, were exported and used in destructive analysis with formal approval by the Mexican authorities (Consejo de Arqueología, form 401.1S.3-2019/1785), and sampling again followed best-practice for such analysis.

### Modern comparative sequences

The 159 modern mitogenomes used for comparison with the archaeological samples were retrieved from GenBank (full list in Table 1). The Y chromosome intron sequences were retrieved from GenBank: DDX3Y intron 1 (GenBank AY928811), DDX3Y intron 7 (GenBank AY928819), UTY intron 19 (GenBank AY936542), ZFY intron 10 (GenBank BOSZFY3), ZFY intron 9 (GenBank AY928823).

### Ancient DNA extraction protocol and sequencing

Specimens were processed at the Florida Museum of Natural History (FLMNH) ancient DNA lab. The DNA extraction protocol followed Yang and colleagues^58^ with modifications designed by the authors^59,60^. All the pre-PCR steps were carried out in a dedicated clean facility free of modern PCR products. The specimens were surface sterilized using a 6% bleach solution. For each extraction approximately 500 mg of bone was removed from the specimen. Subsamples were frozen using liquid nitrogen and then crushed into a fine powder. The resultant bone powder was then placed into a 1000 µL of EDTA-based extraction buffer containing sodium dodecyl sulfate, dithiothreitol, and proteinase K. After a 24 h incubation period at 65 °C, the supernatant was concentrated then transferred into QIAquick columns to remove the DNA from the other components using Qiagen PB and PE buffers. The DNA was then eluted in 25 µL of Qiagen EB buffer. In parallel, a negative control was run following the same steps but without any bone powder to monitor contamination during the DNA extraction. DNA quantification of the sample and negative control were performed with a Qubit. Qubit quantifications for the negative controls in each assay did not detect any DNA.

For the recovery of mitochondrial genome data, the DNA extractions were sent to Rapid Genomics (Gainesville, Florida) for library preparation, mtDNA enrichment, and sequencing. DNA libraries were generated using Swift (now IDT) Methyl-Seq Library Preparation kits used to prepare genomic DNA for downstream steps, but excluding the bisulfite conversion step.This kit uses a uracil-tolerant polymerase and performs well with degraded and low-yield samples. This method was recommended by the vendor as being particularly efficient in converting short, single-stranded fragments of uracil-containing DNA into NGS libraries (www.idtdna.com). We performed 15 PCR cycles for the indexing PCR step). The overall approach has been previously implemented with success in prior analyses of fossil and historical samples^60,61^ The SPRI bead cleanup ratios were modified to retain lower molecular weight fragments : post-extension SPRI ratio 1.8; post-ligation SPRI ratio 1.6; post-PCR SPRI ratio 1.6.

Rapid Genomics designed RNA bait kits for the enrichments using 12,000 probes based on the domestic cattle (*Bos taurus*) mitogenome (NC_006853.1). Enrichments were performed on the library utilizing all the library product or up to 500 ng following Rapid Genomics customized workflow. Two rounds of hybridization were performed on each sample at 60 °C for 48 h. After each hybridization step and clean up the sample was subjected to 15 cycles of PCRs. The enriched samples were sequenced on an Illumina MiSeq sequencer.

The eight samples for the Y chromosome analysis included the following samples: Puerto Real 4, Puerto Real 6, Merida 12, Merida 15, Merida 16, Mexico Justo Sierra 20, Mexico Bellas Artes 22, Mexico Bellas Artes 24. They were processed in the FLMNH ancient DNA lab. After extraction following the same protocol described above, libraries were prepared using Swift Methyl-Seq Library Preparation Kit. The resulting libraries went through enrichment using custom RNA baits designed by Arbor Biosciences based on the sequences of five *Bos taurus* Y chromosome introns: DDX3Y intron 1 (GenBank AY928811), DDX3Y intron 7 (GenBank AY928819), UTY intron 19 (GenBank AY936542), ZFY intron 10 (GenBank BOSZFY3), ZFY intron 9 (GenBank AY928823). Two rounds of hybridization were performed on each sample at 60 °C for 48 h. After each enrichment and cleanup, the sample underwent 15 PCR cycles. The enriched libraries were sequenced on an Illumina NovaSeq 6000 sequencer at the University of Florida Interdisciplinary Center for Biotechnology Research (UF | ICBR).

### NGS reads processing

The quality of the reads from the sequencing was first assessed using FASTQC^62^. After adapter removal and quality trimming using the Fastp^63^, the 3’ and 5’ reads were imported and paired in Geneious Prime 2021.1.1. After removing the duplicates in Geneious, we mapped the reads to the cattle mitochondrial reference genome (GenBank NC_006853.1), using the Geneious Prime mapper algorithm set on “Custom sensitivity” with a minimum mapping quality of 30, allowing for only 5% mismatch between reads, allowing 5% gaps per mapped read. The Geneious Prime algorithm maps reads to the reference up to 5 times. A contiguous mitochondrial genome sequence was generated by Geneious with a threshold of 75% and quality set on “Highest”. Geneious has proven to provide robust results in mapping and assembly tasks for a variety of ancient degraded samples^59–61^.

The processing of the Y chromosome NGS reads followed the same pipeline using published sequences of the five *Bos taurus* Y chromosome introns (DDX3Y intron 1 (GenBank AY928811), DDX3Y intron 7 (GenBank AY928819), UTY intron 19 (GenBank AY936542), ZFY intron 10 (GenBank BOSZFY3), ZFY intron 9 (GenBank AY928823) as references. After generation of the consensus sequences, the resulting FASTA files were then aligned with sequences of modern individuals of known provenience. We finally processed the FASTA alignment file through GBlocks with default settings to remove ambiguously aligned sequences^64^.

### DNA degradation assessment

The damage patterns of the mitogenome and Y chromosome introns assemblies were assessed using MapDamage^65^. The DNA damage patterns for the mapped sequences are consistent with those of ancient DNA (Fig. S2 to S22): at the 5' ends of sequences, there is a surplus of C-to-T misincorporations and complementary G-to-A misincorporations at the 3' termini, which are caused by increased cytosine deamination in single-stranded 5'-overhanging ends^66^. Additionally, there is an abundance of purines found in the genomic coordinate just prior to the start of sequencing, which is an indication of post-mortem depurination and subsequent strand fragmentation.

### Median-joining networks

The FASTA alignments of the archaeological sequences with 159 modern reference sequences (Table S1) were processed using PopART (Population Analysis with Reticulate Trees) to obtain an MJ network^67^. This approach illustrates the relationships between taxa based on Kruskal's algorithm for generating minimum spanning trees and Farris's maximum-parsimony (MP) heuristic algorithm.

### Maximum-likelihood (ML) phylogeny

A phylogeny was built using an alignment containing archaeological sequences and modern sequences and RAxML with the GTRCAT model of rate heterogeneity and 10,000 bootstrap replicates^46^. The mitogenome data were partitioned based on the annotations imported from the reference sequence into coding (CDS), non-coding (introns), rRNA, and tRNA regions to produce a phylogeny using different estimates for each of these regions. We used *Bos indicus* reference mitogenomes as outgroups.

## Supplementary Information

Supplementary Information.

## Data Availability

The datasets generated and analyzed during the current study are available in the GenBank repository (Accession # OP858992-OP859012) and in the Sequence Read Archive (SRA Bioproject # PRJNA903372, https://www.ncbi.nlm.nih.gov/sra/PRJNA903372).
