# Supplementary material for: Ancient DNA confirms diverse origins of early post-Columbian cattle in the Americas
Source: Sci Rep. 2023 Aug 1;13:12444. doi: 10.1038/s41598-023-39518-3 (PMC10394069; doi:10.1038/s41598-023-39518-3)
Supplement: Supplementary file 1 — Supplementary Information. [file 41598_2023_39518_MOESM1_ESM.docx]

**Supporting Information for**

Ancient DNA confirms diverse origins of early Post-Columbian cattle in the Americas

Nicolas Delsol^1^*, Brian J. Stucky^1,2^, Jessica A. Oswald^1,3^, Charles R. Cobb^1^, Kitty F. Emery^1^, Robert Guralnick^1^

^1^ Florida Museum of Natural History, University of Florida, Gainesville, FL 32611 Florida, USA

^2^ Agricultural Research Service, U.S. Department of Agriculture, Beltsville, MD, USA

^3^ Biology Department, University of Nevada, Reno, Reno, NV 89557, USA

* Corresponding author

Nicolas Delsol

**Email:**  [ndelsol@ufl.edu](mailto:ndelsol@ufl.edu)

**This PDF file includes:**

Figure S1 to S22

Table S1 to S22


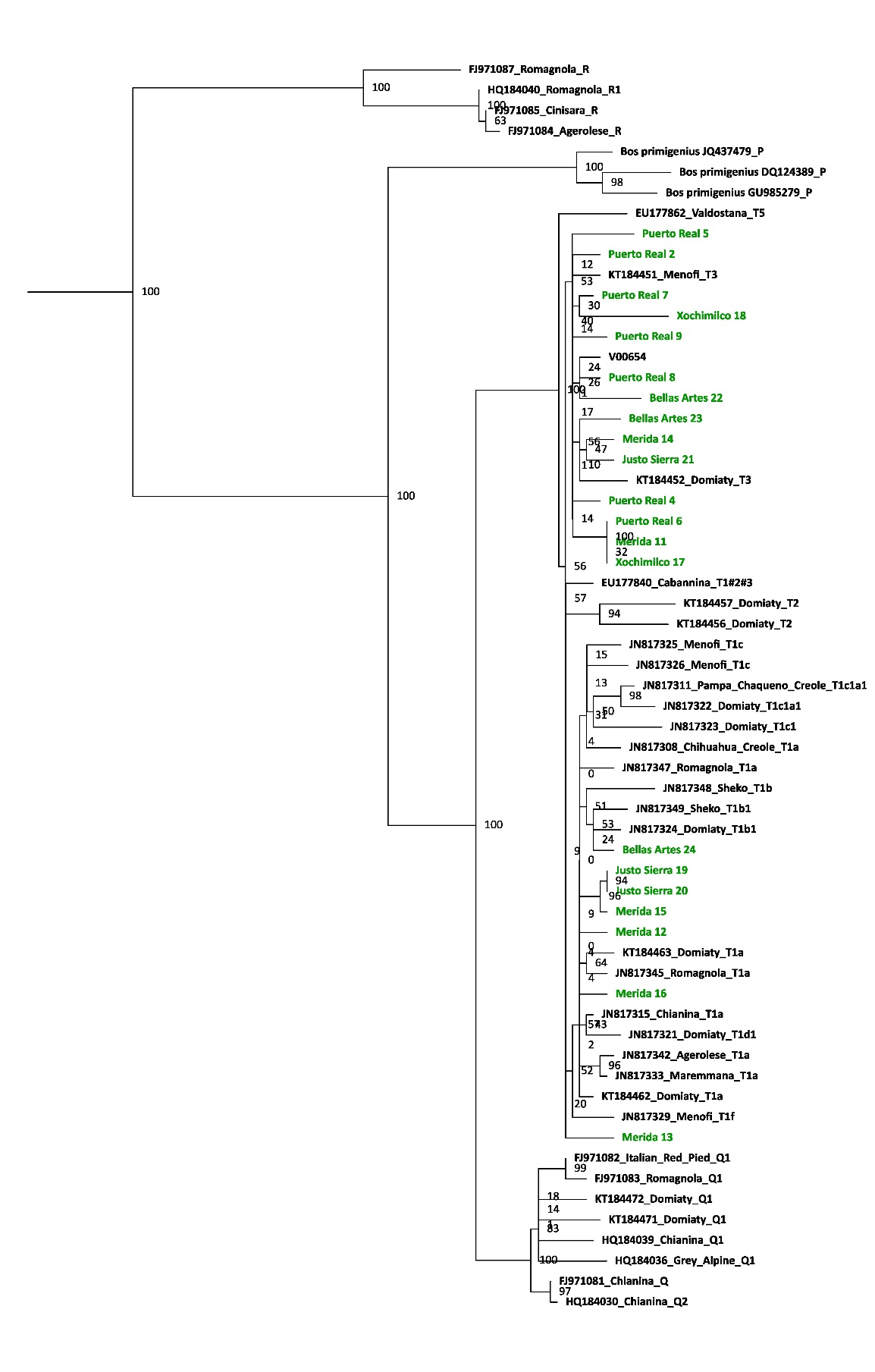
**Fig. S1.** Maximum likelihood tree for 21 archaeological and 42 modern cattle mitogenomes realized with RAxML using the GTRCAT model of rate heterogeneity and 10,000 bootstrap replicates. The outgroup (*Bos indicus*, GenBank # NC005971 and EU177869) is not represented on the figure for greater legibility.

R0121_PR2_align


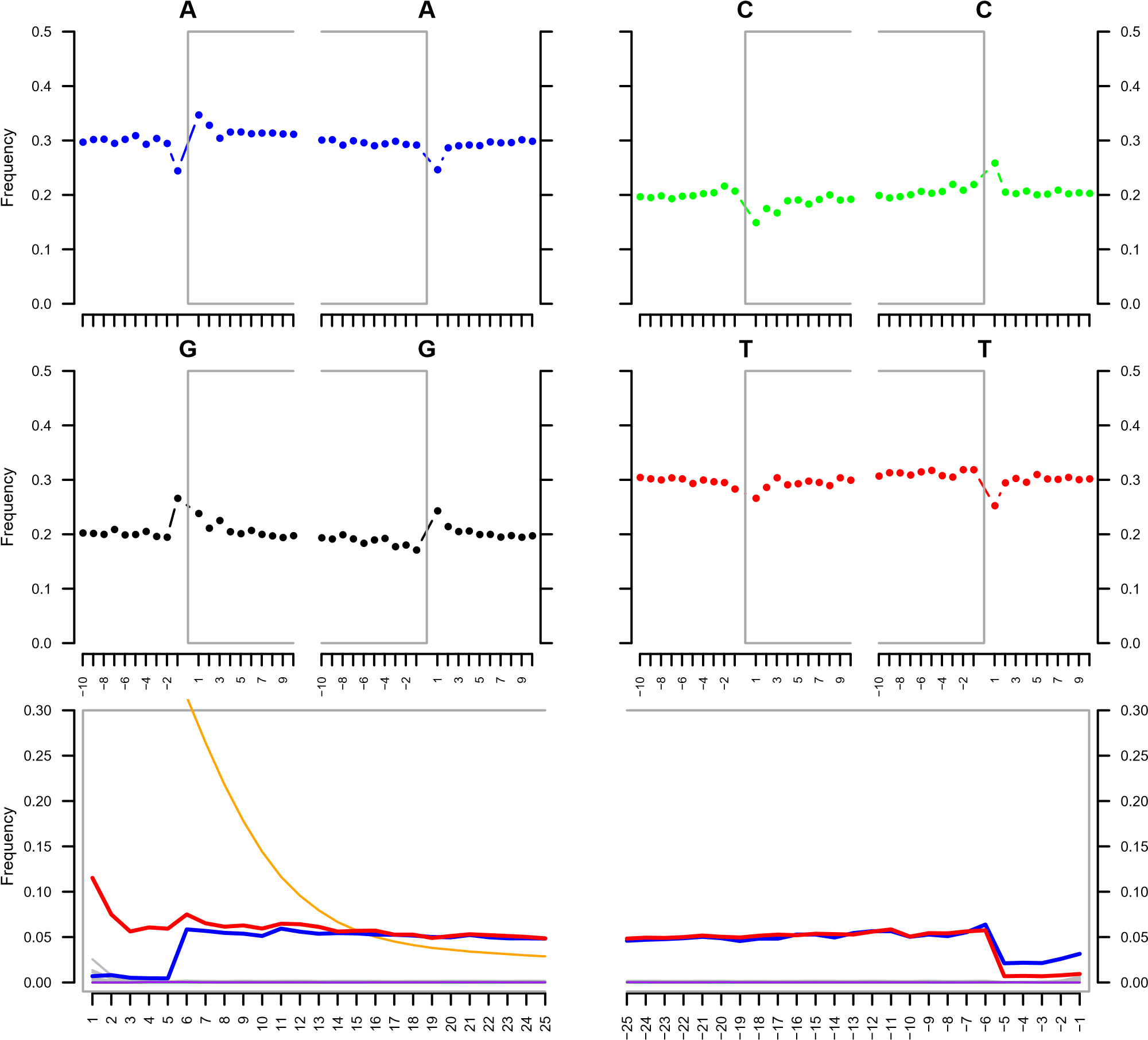


**Fig. S2.** MapDamage fragment misincorporation plot for the Puerto Real 2 sample

R0121_PR4_align


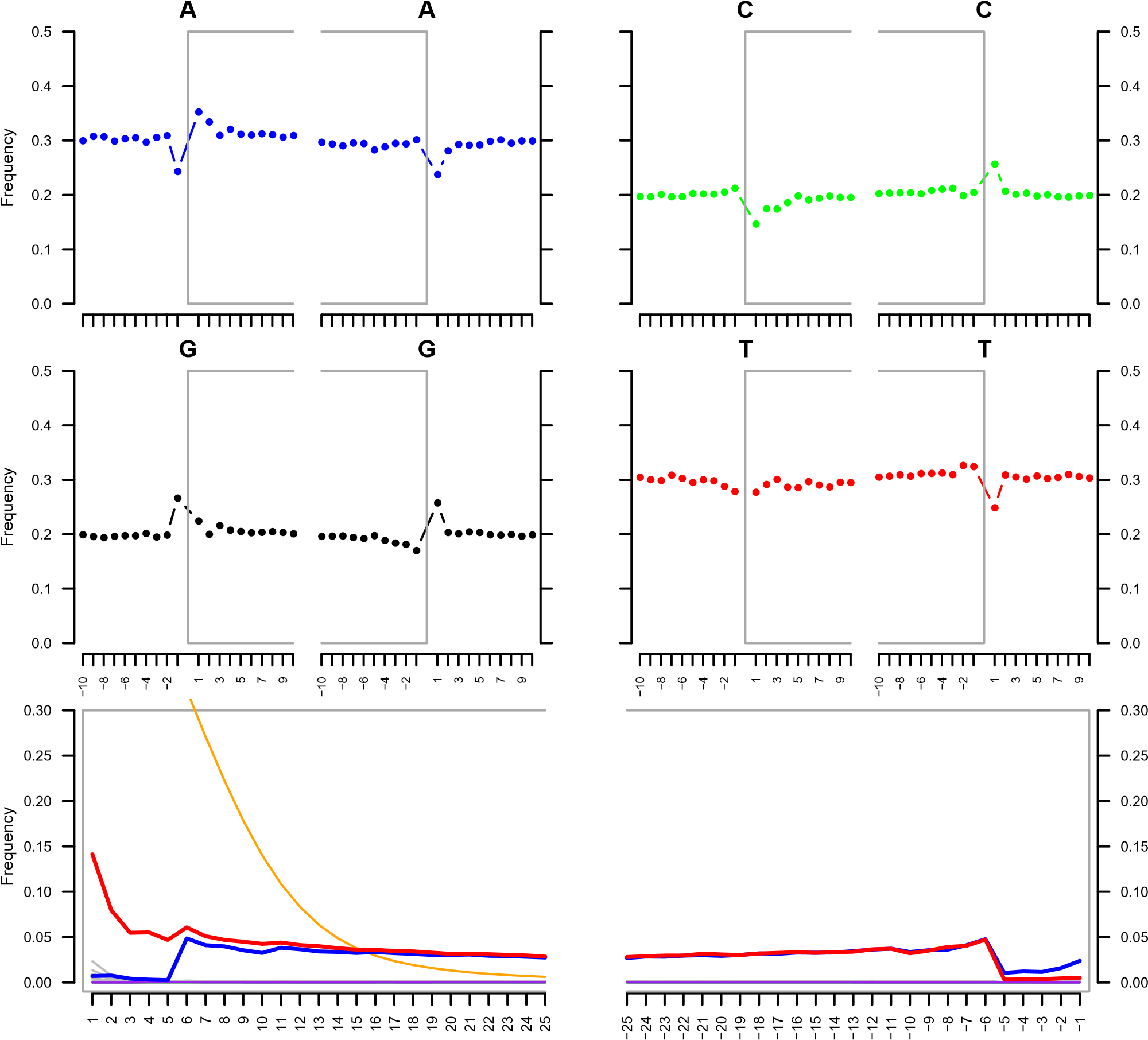


**Fig. S3.** MapDamage fragment misincorporation plot for the Puerto Real 4 sample

R0121_PR5_align


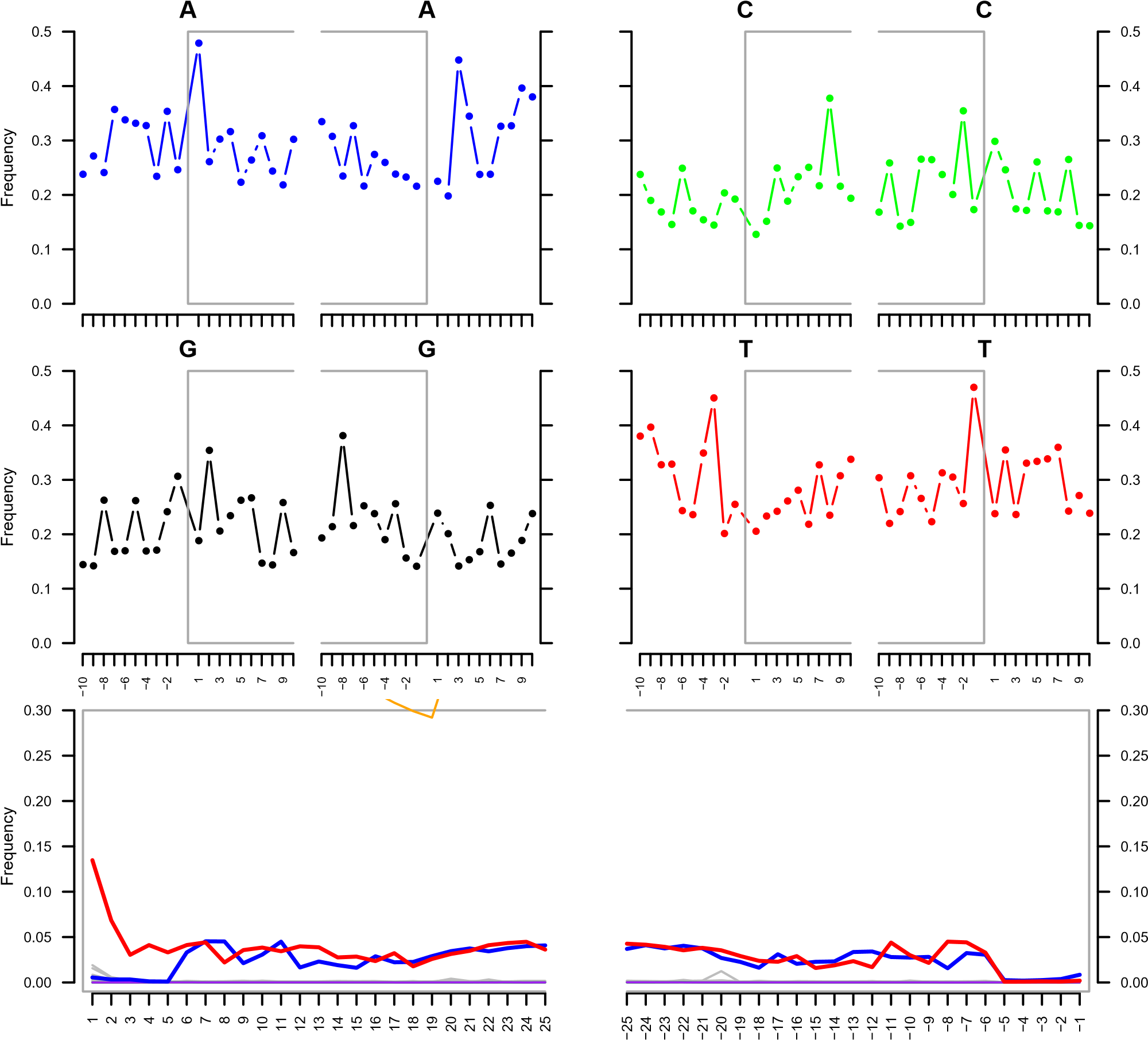


**Fig. S4.** MapDamage fragment misincorporation plot for the Puerto Real 5 sample

R0121_PR6_align


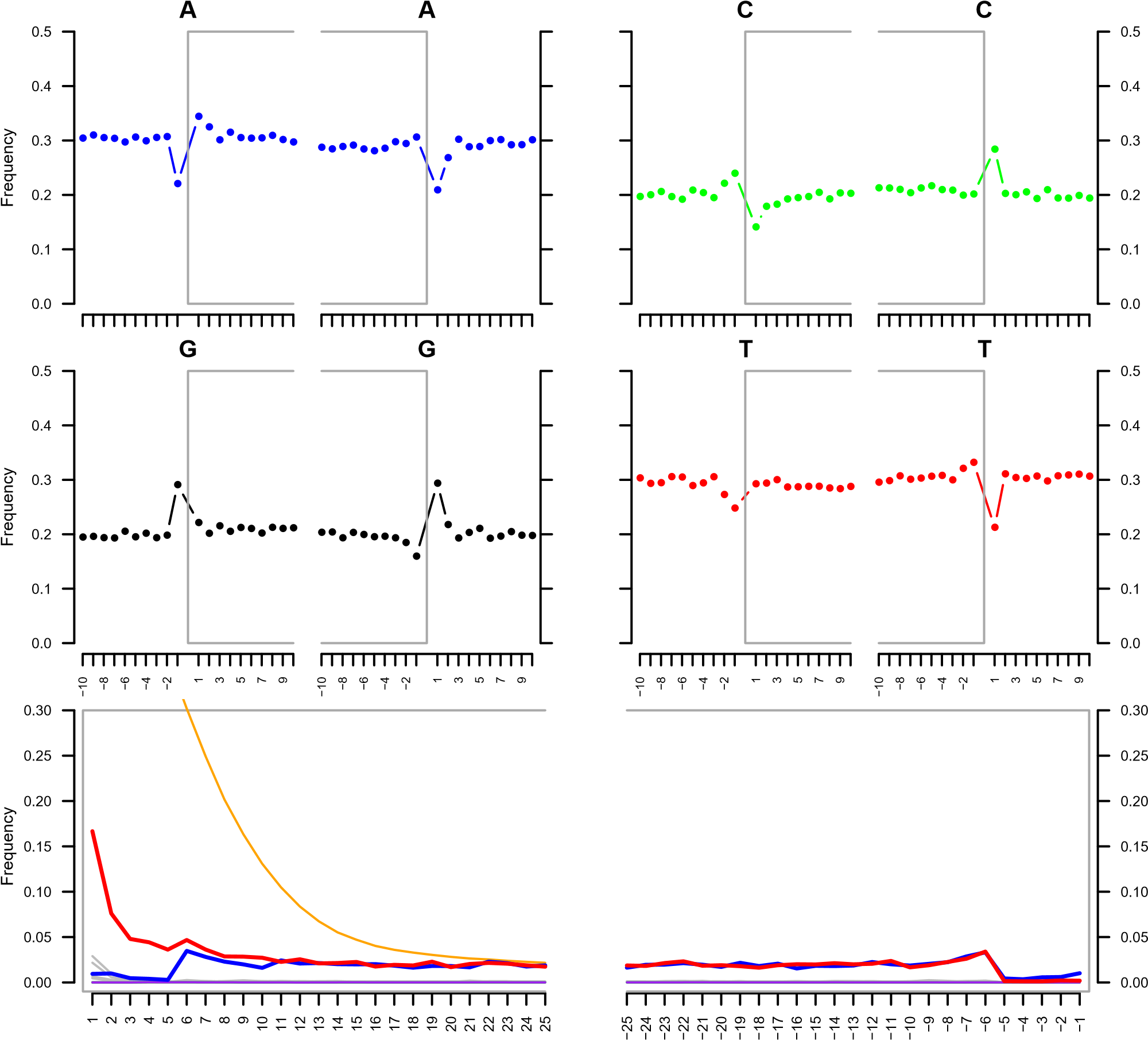


**Fig. S5.** MapDamage fragment misincorporation plot for the Puerto Real 6 sample

R0121_PR7_align


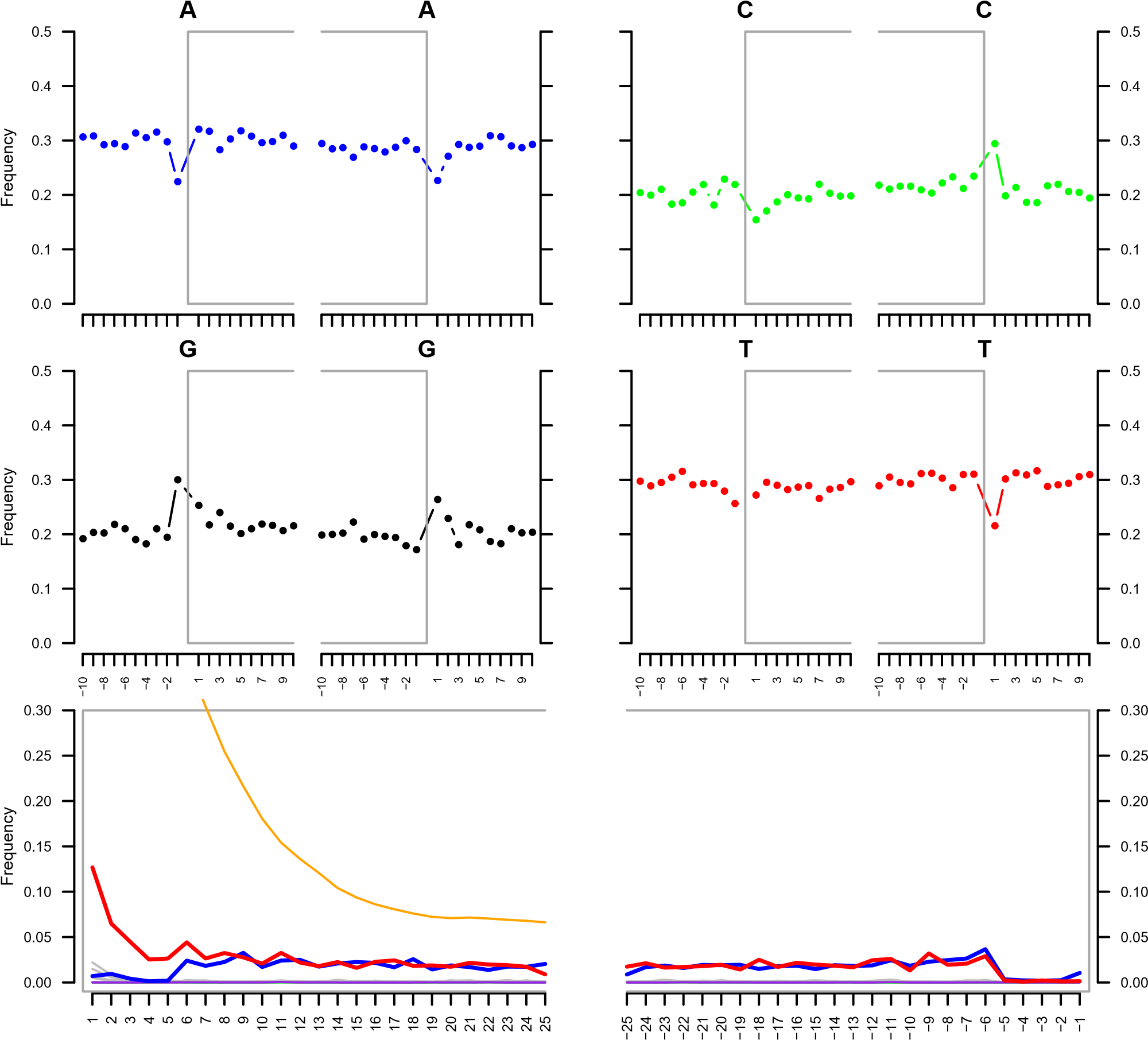


**Fig. S6.** MapDamage fragment misincorporation plot for the Puerto Real 7 sample

R0121_PR8_align


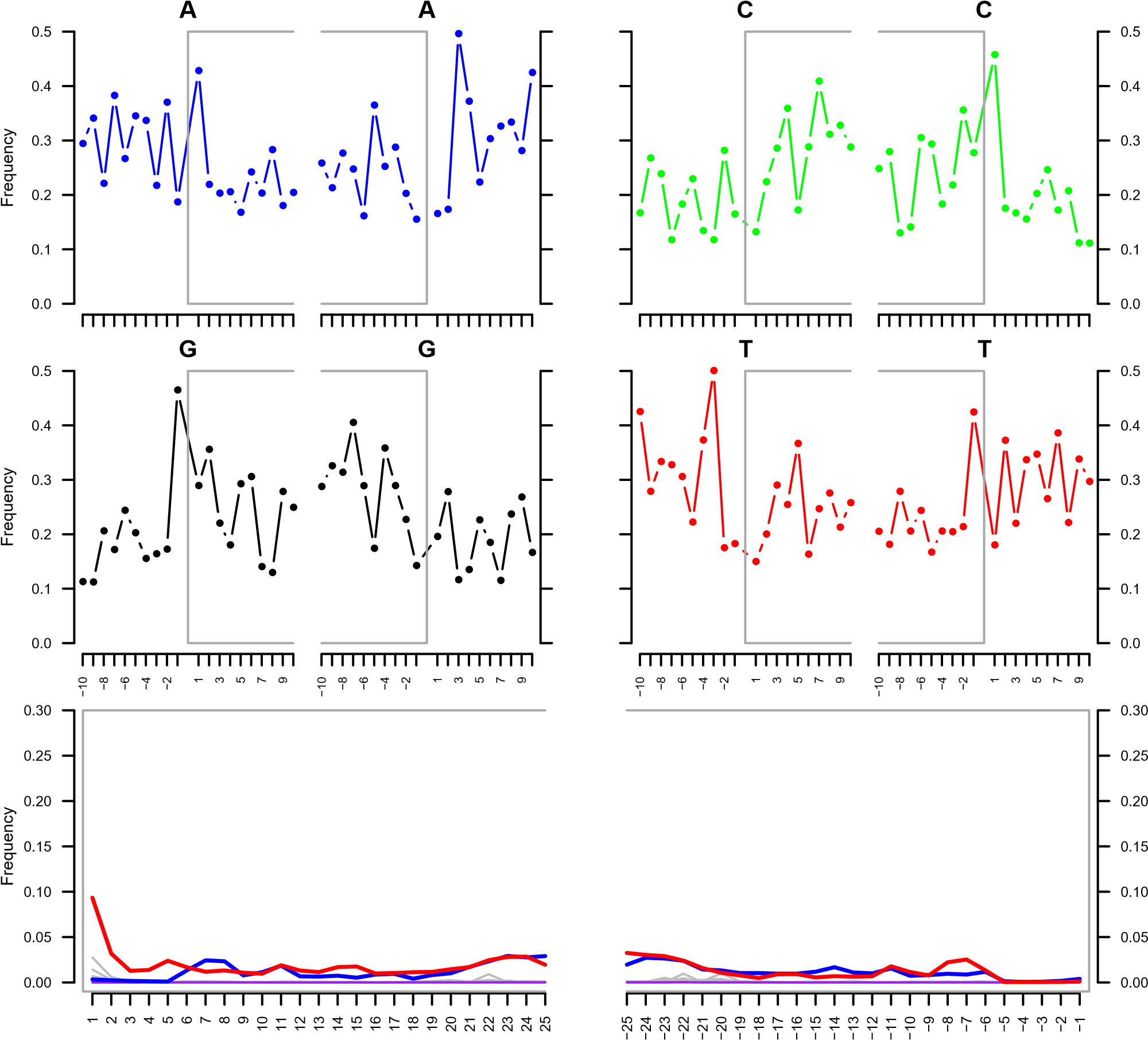


**Fig. S7.** MapDamage fragment misincorporation plot for the Puerto Real 8 sample

R0121_PR9_align


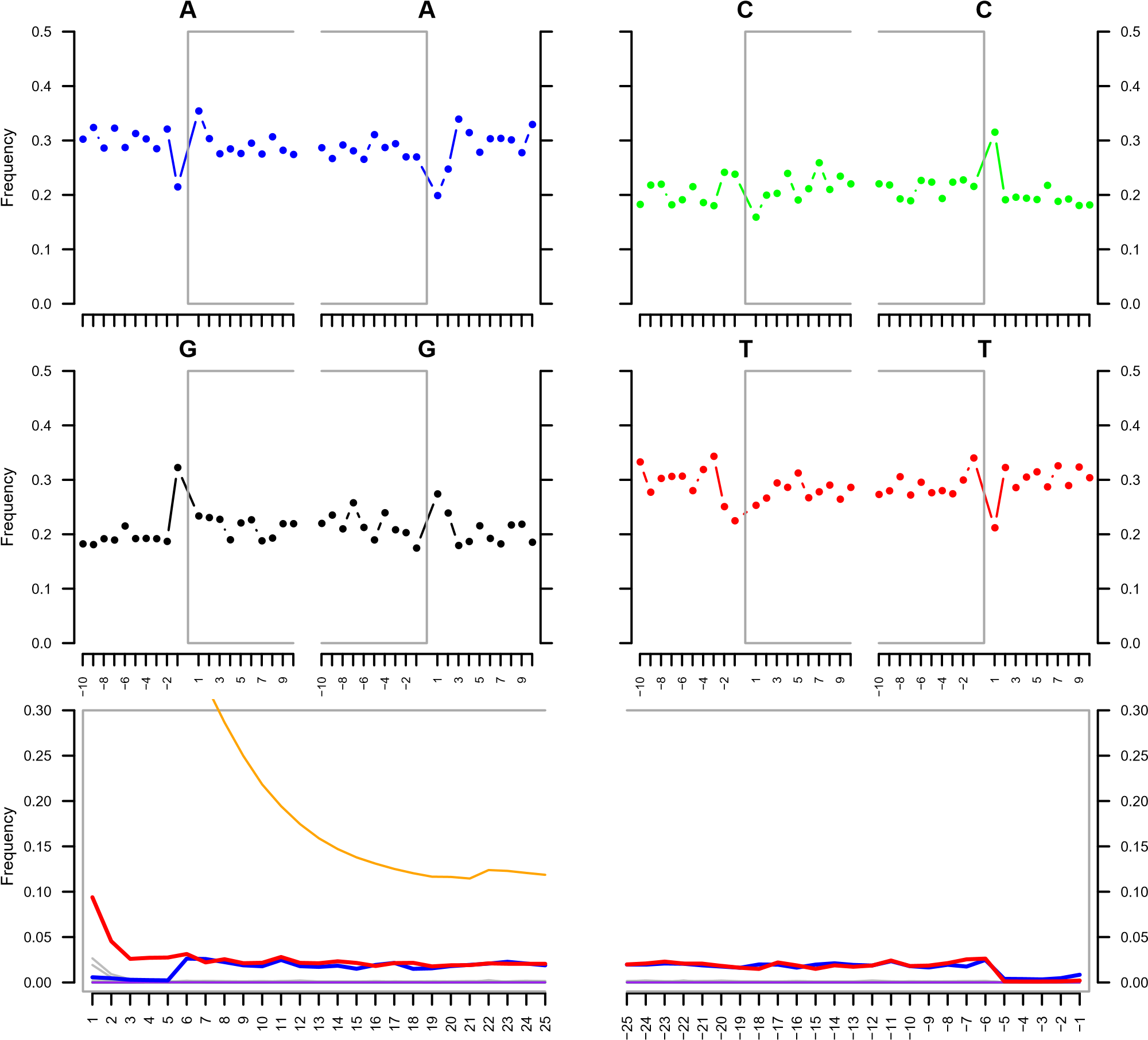


**Fig. S8.** MapDamage fragment misincorporation plot for the Puerto Real 9 sample

R0121_YUC11_align


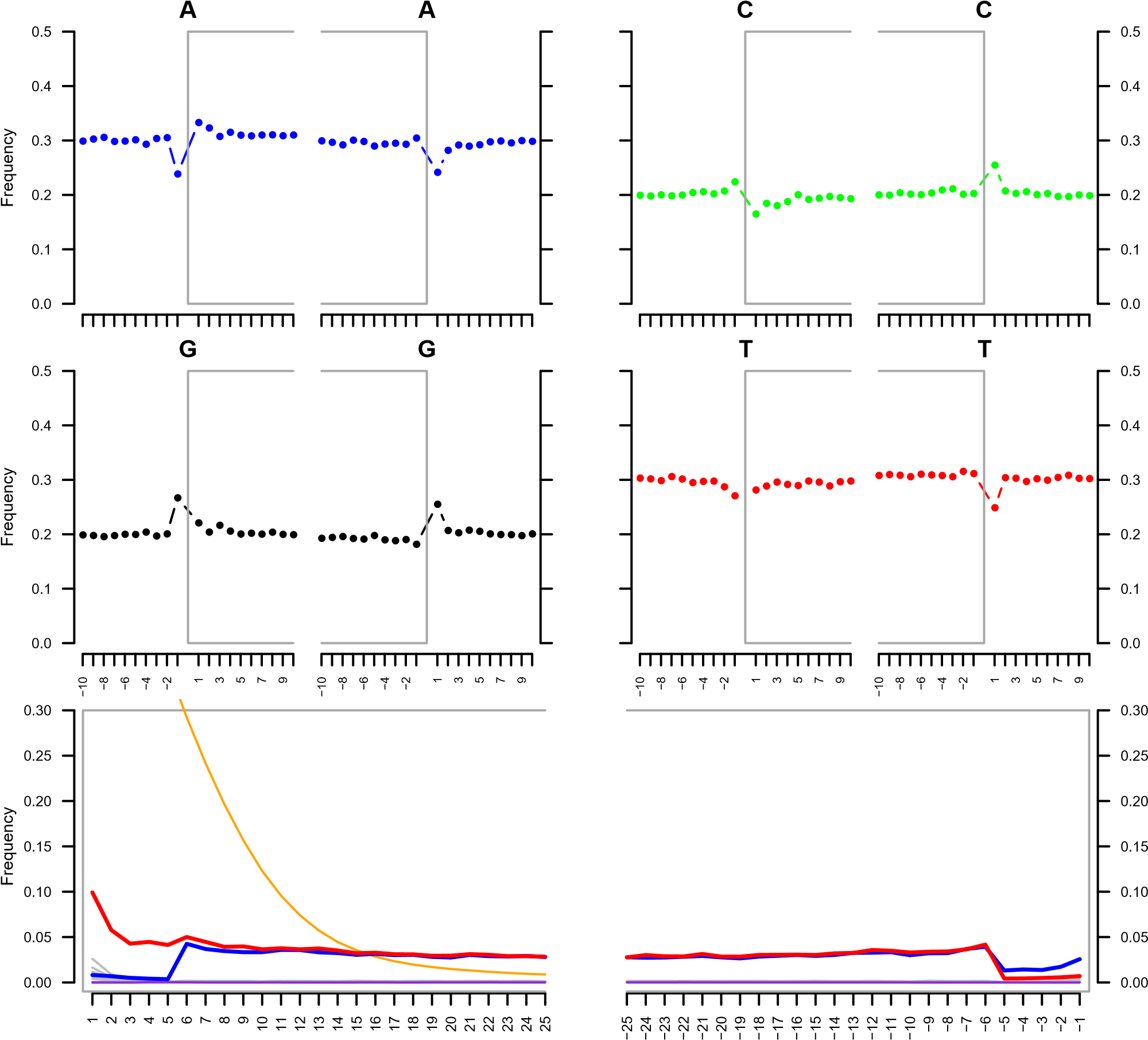


**Fig. S9.** MapDamage fragment misincorporation plot for the Merida 11 sample

R0121_YUC12_align


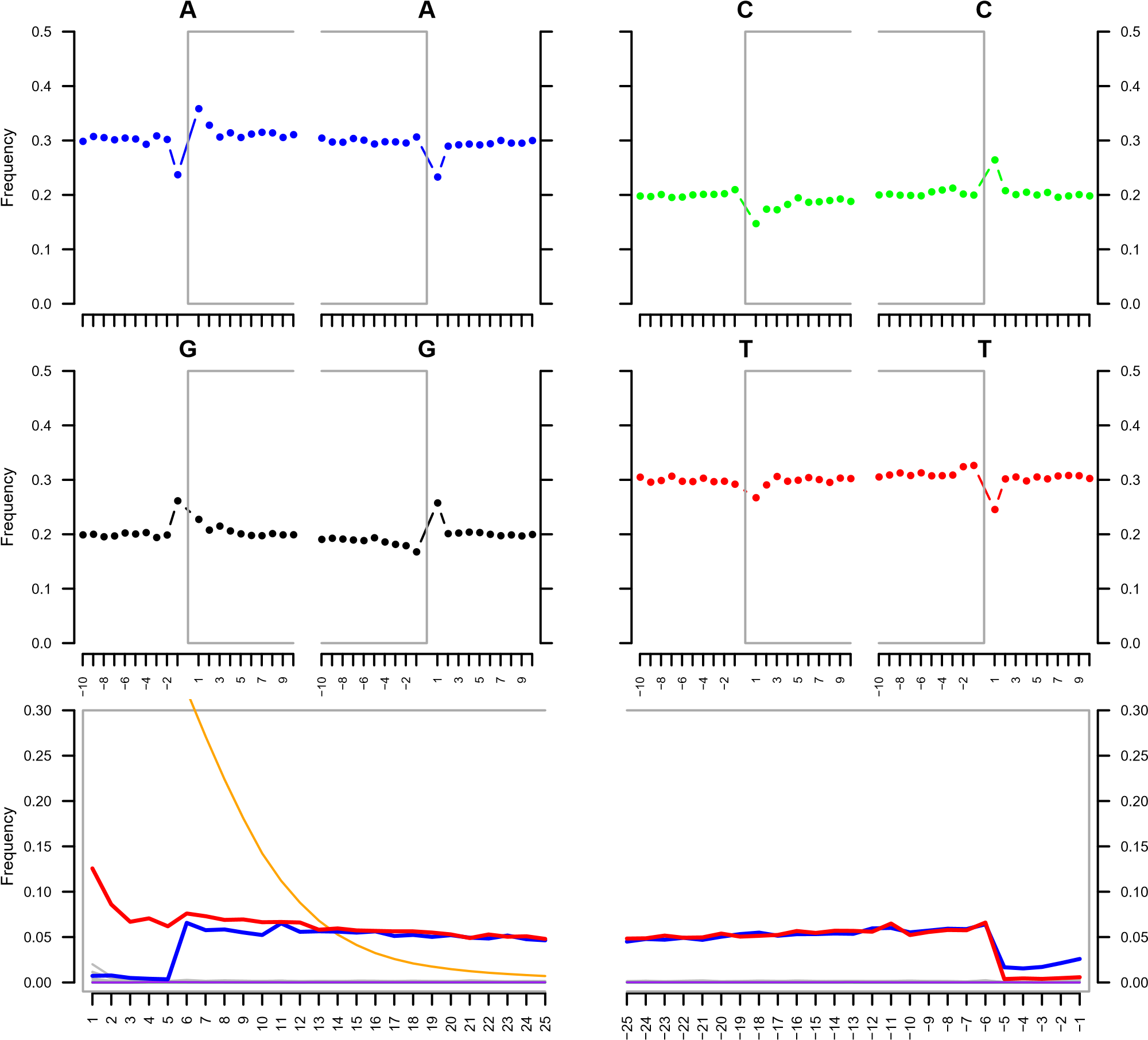


**Fig. S10.** MapDamage fragment misincorporation plot for the Merida 12 sample

R0121_YUC13_align


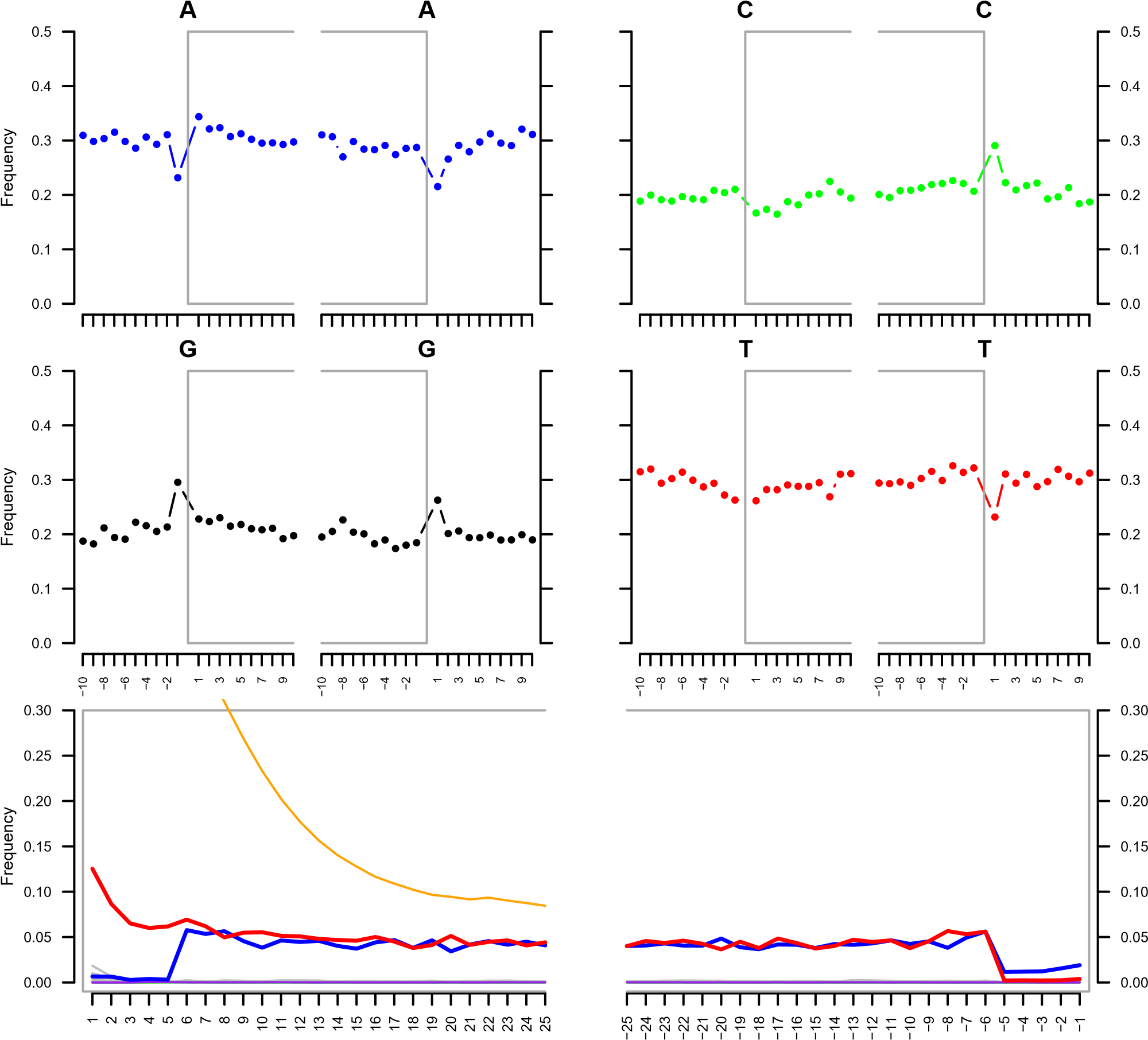


**Fig. S11.** MapDamage fragment misincorporation plot for the Merida 13 sample

R0121_YUC14_align


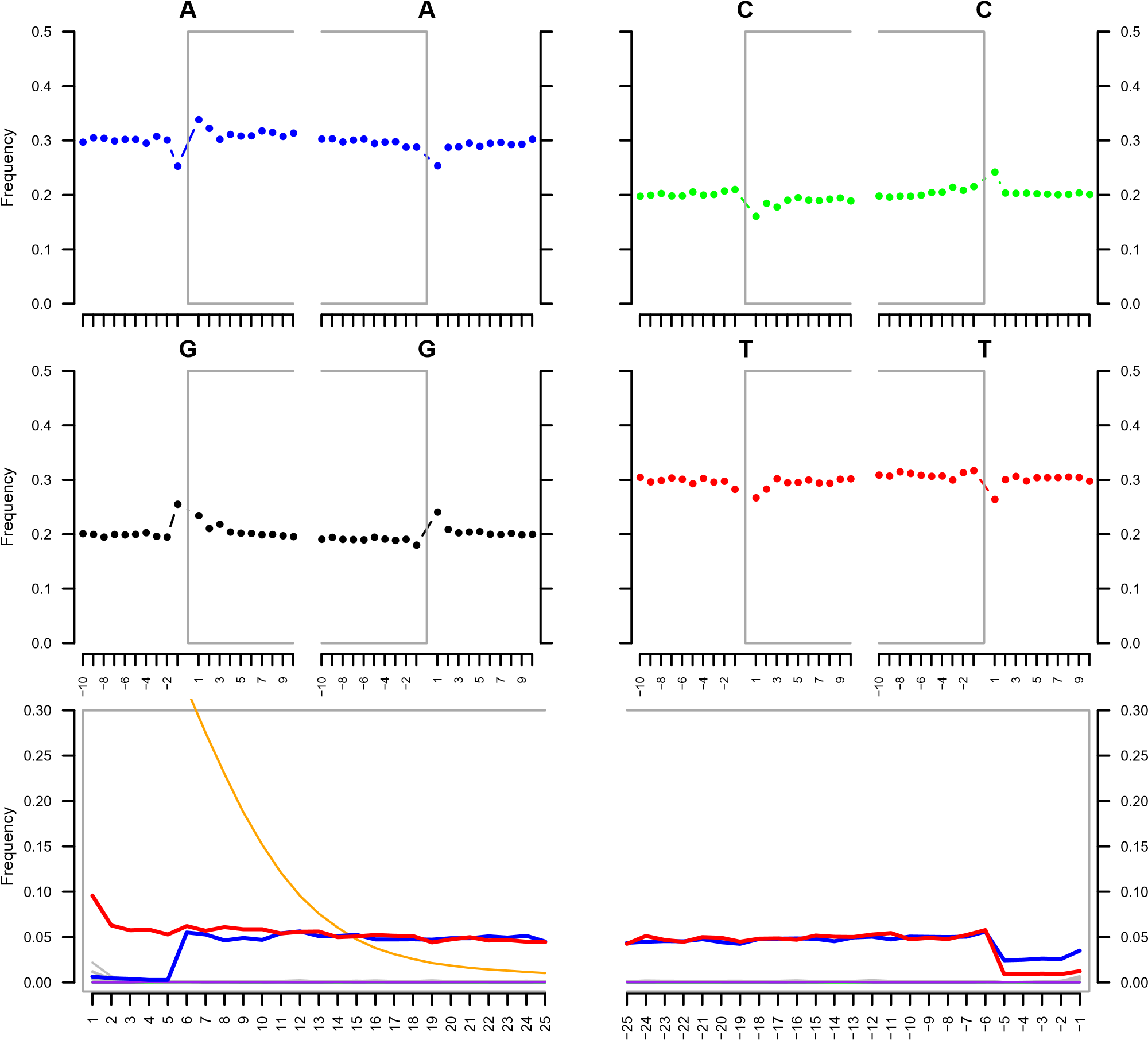


**Fig. S12.** MapDamage fragment misincorporation plot for the Merida 14 sample

R0121_YUC15_align


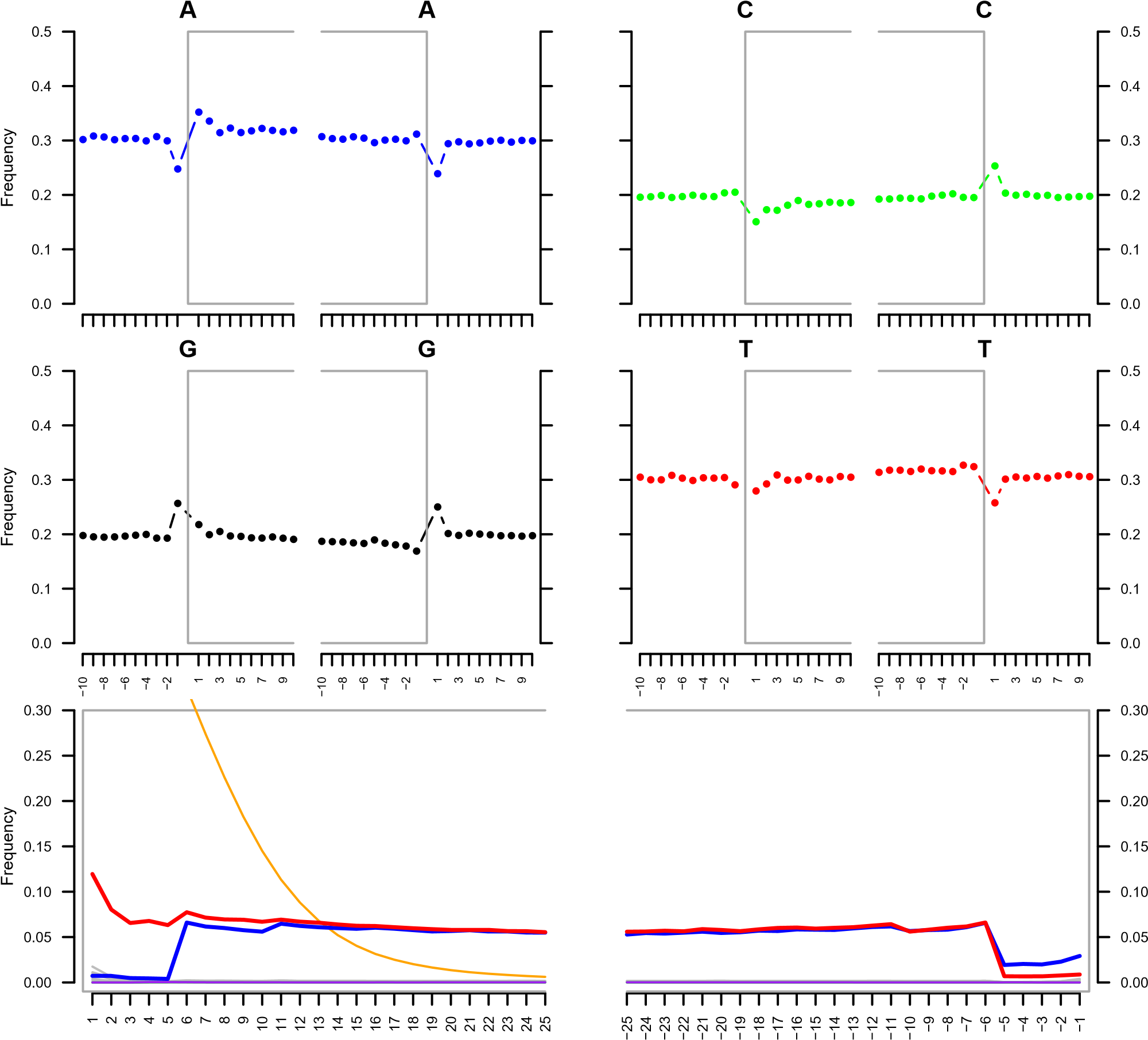


**Fig. S13.** MapDamage fragment misincorporation plot for the Merida 15 sample

R0121_YUC16_align


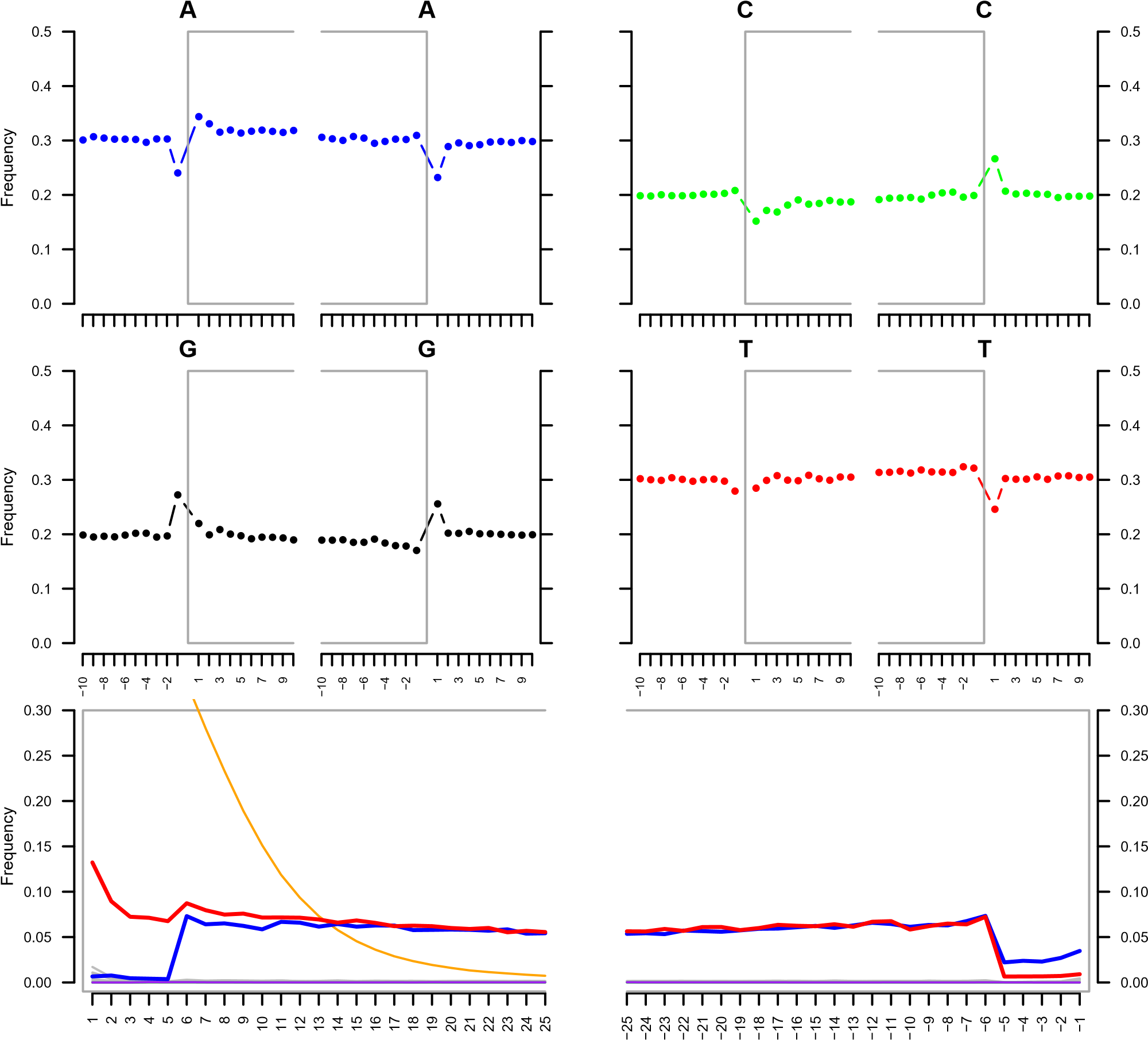


**Fig. S14.** MapDamage fragment misincorporation plot for the Merida 16 sample

R0121_MXXO17_align


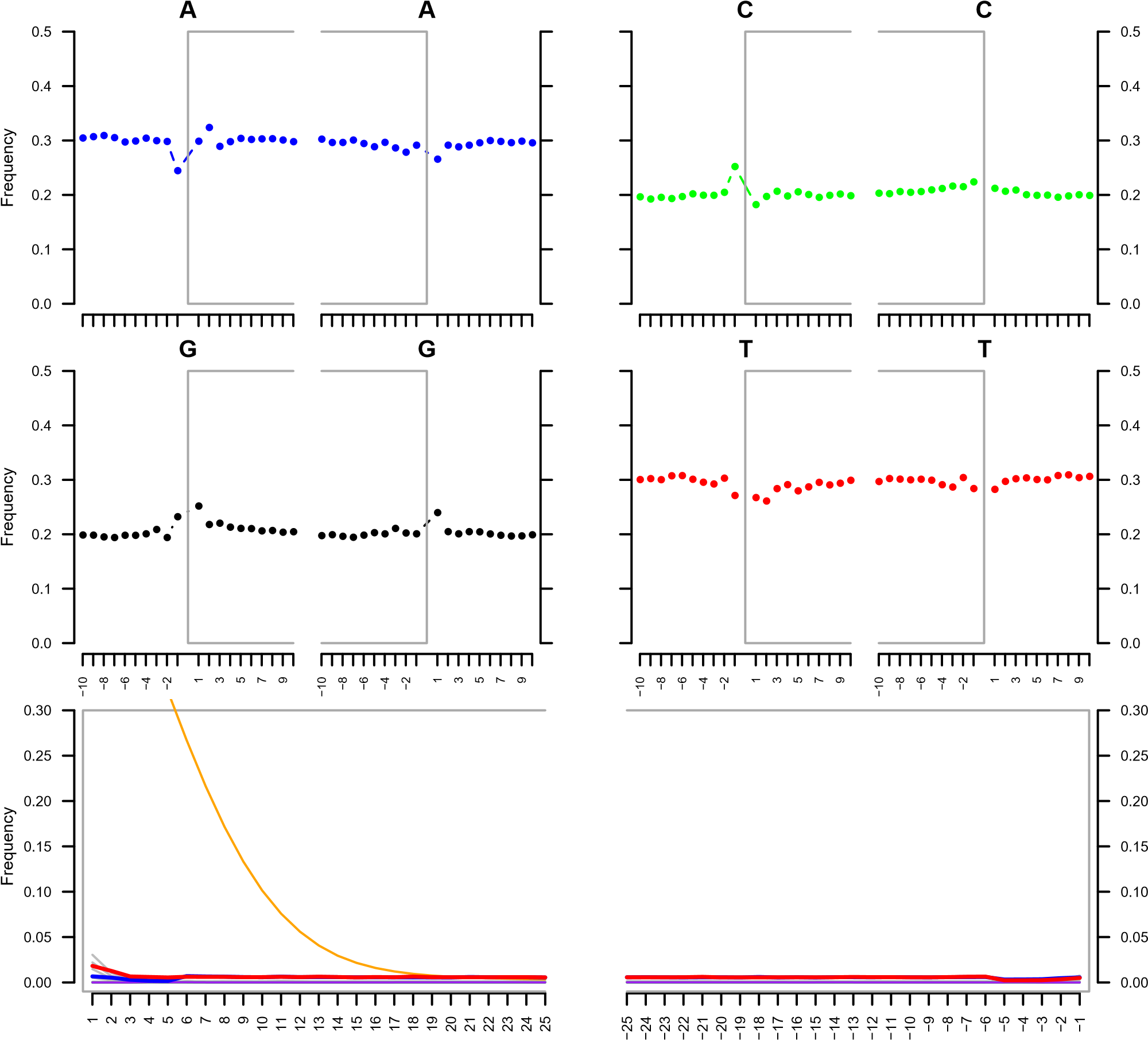


**Fig. S15.** MapDamage fragment misincorporation plot for the Mexico Xochimilco 17 sample

R0121_MXXO18_align


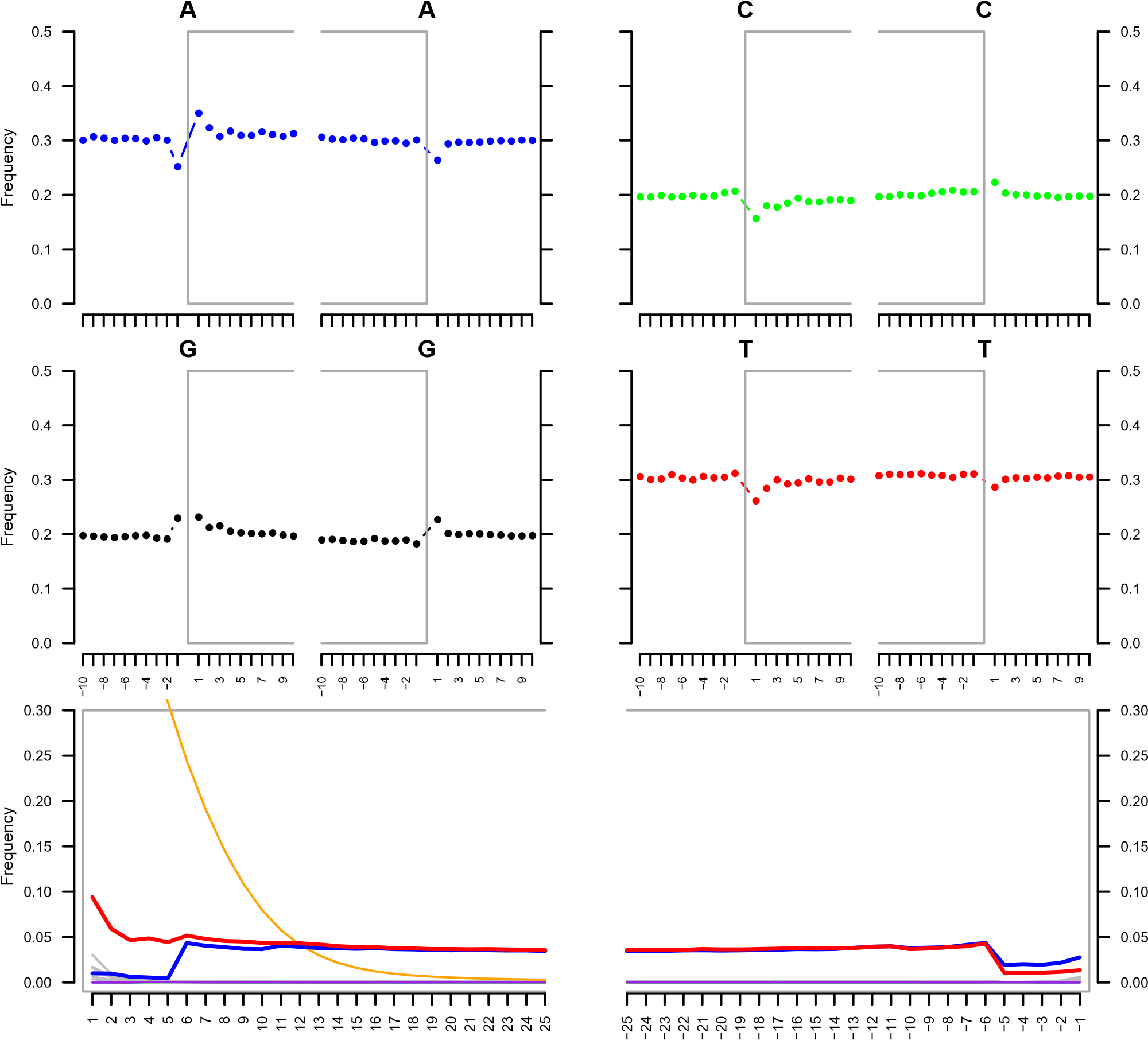


**Fig. S16.** MapDamage fragment misincorporation plot for the Mexico Xochimilco 18 sample

R0121_MXJS19_align


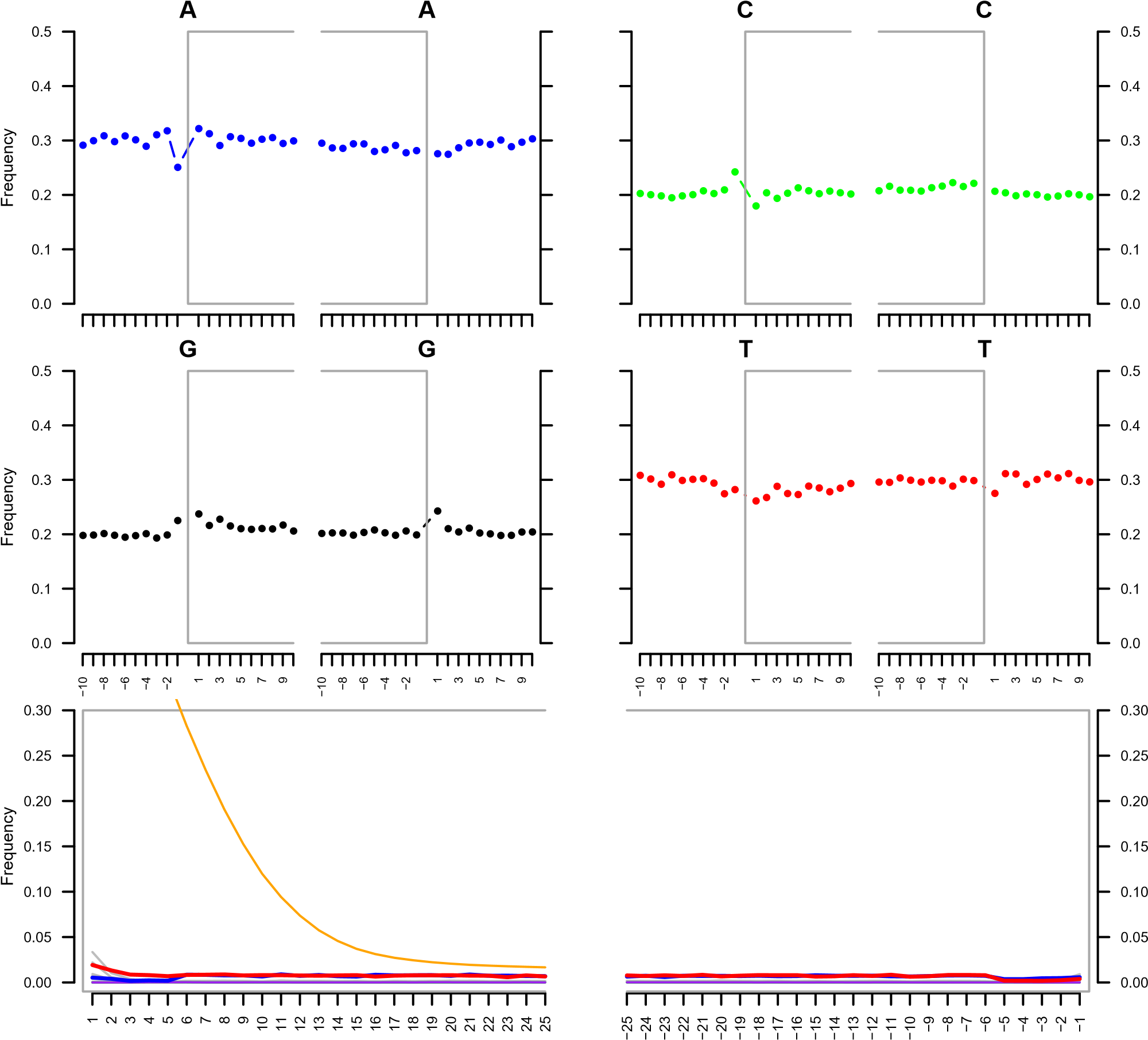


**Fig. S17.** MapDamage fragment misincorporation plot for the Mexico Justo Sierra 19 sample

R0121_MXJS20_align


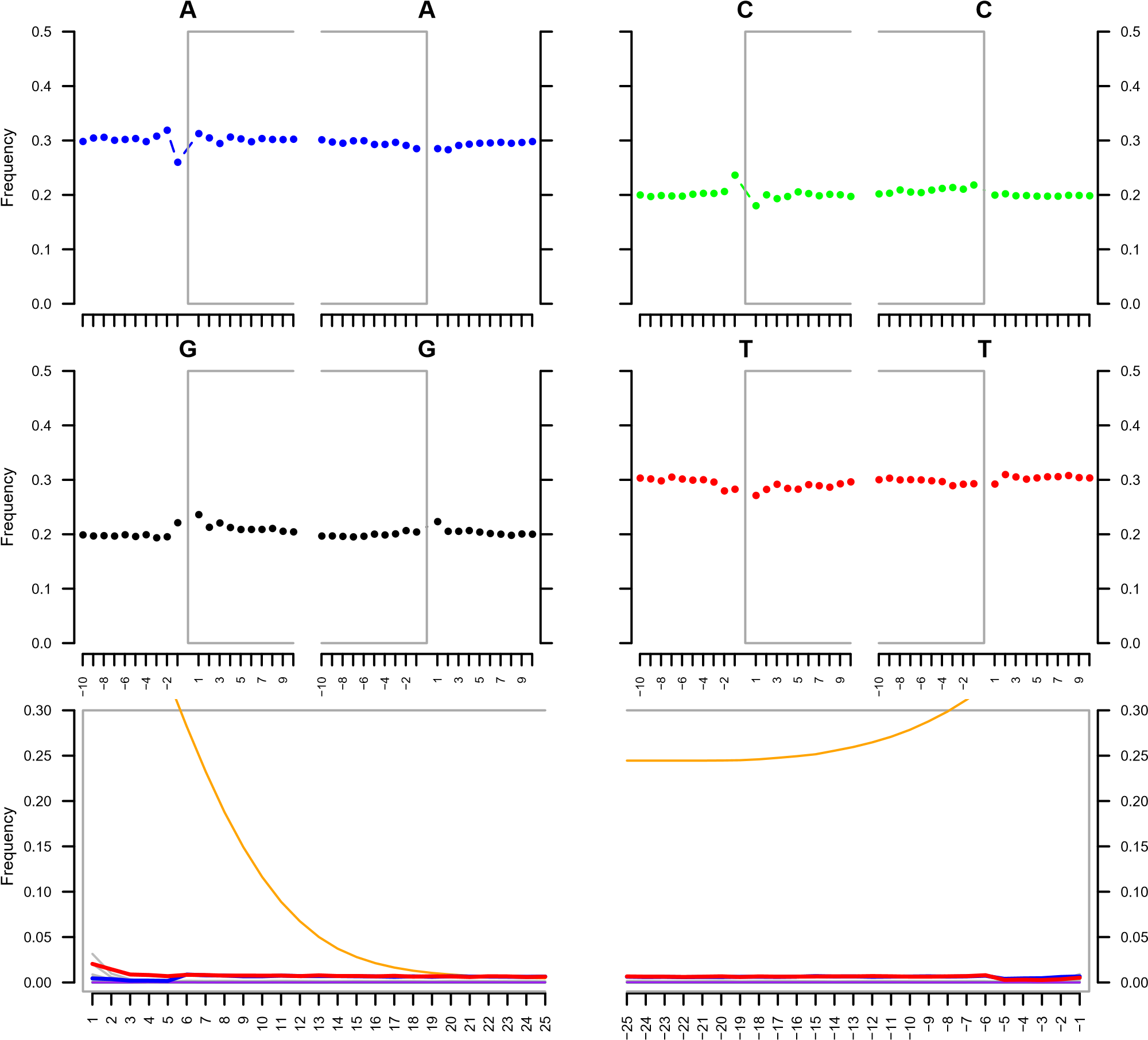


**Fig. S18.** MapDamage fragment misincorporation plot for the Mexico Justo Sierra 20 sample

R0121_MXJS21_align


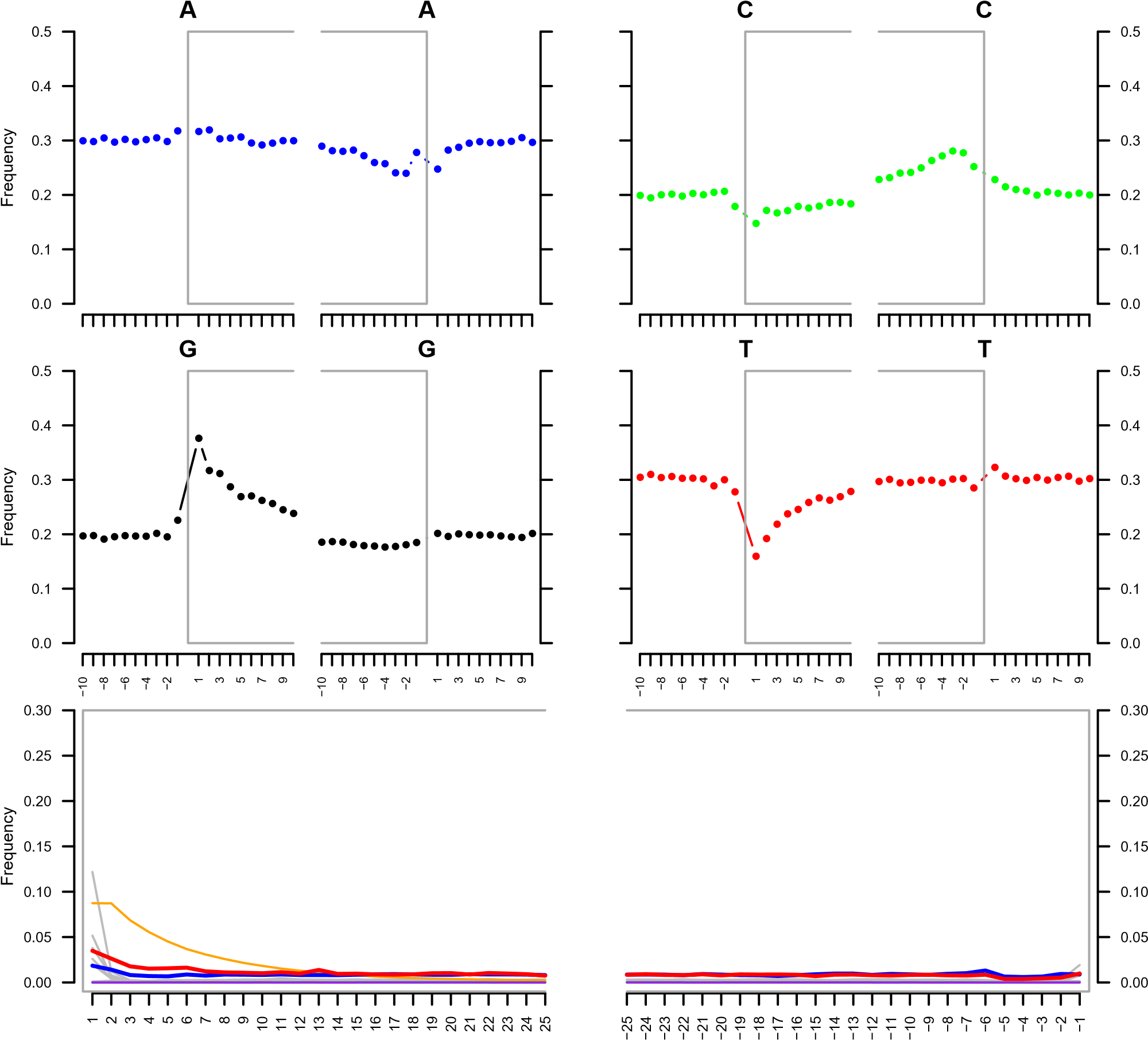


**Fig. S19.** MapDamage fragment misincorporation plot for the Mexico Justo Sierra 21 sample

R0121_MXBA22_align


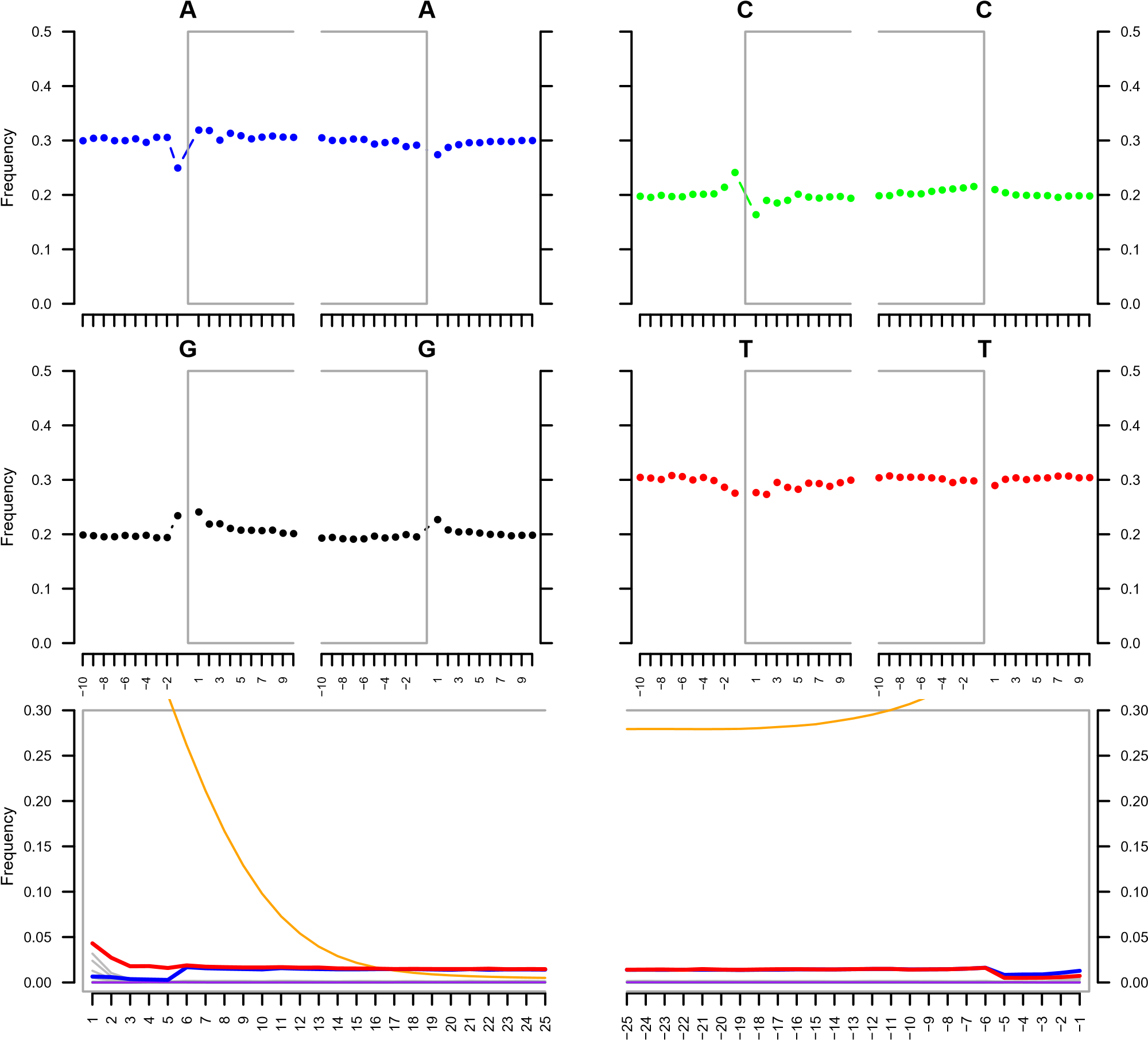


**Fig. S20.** MapDamage fragment misincorporation plot for the Mexico Bellas Artes 22 sample

R0121_MXBA23_align


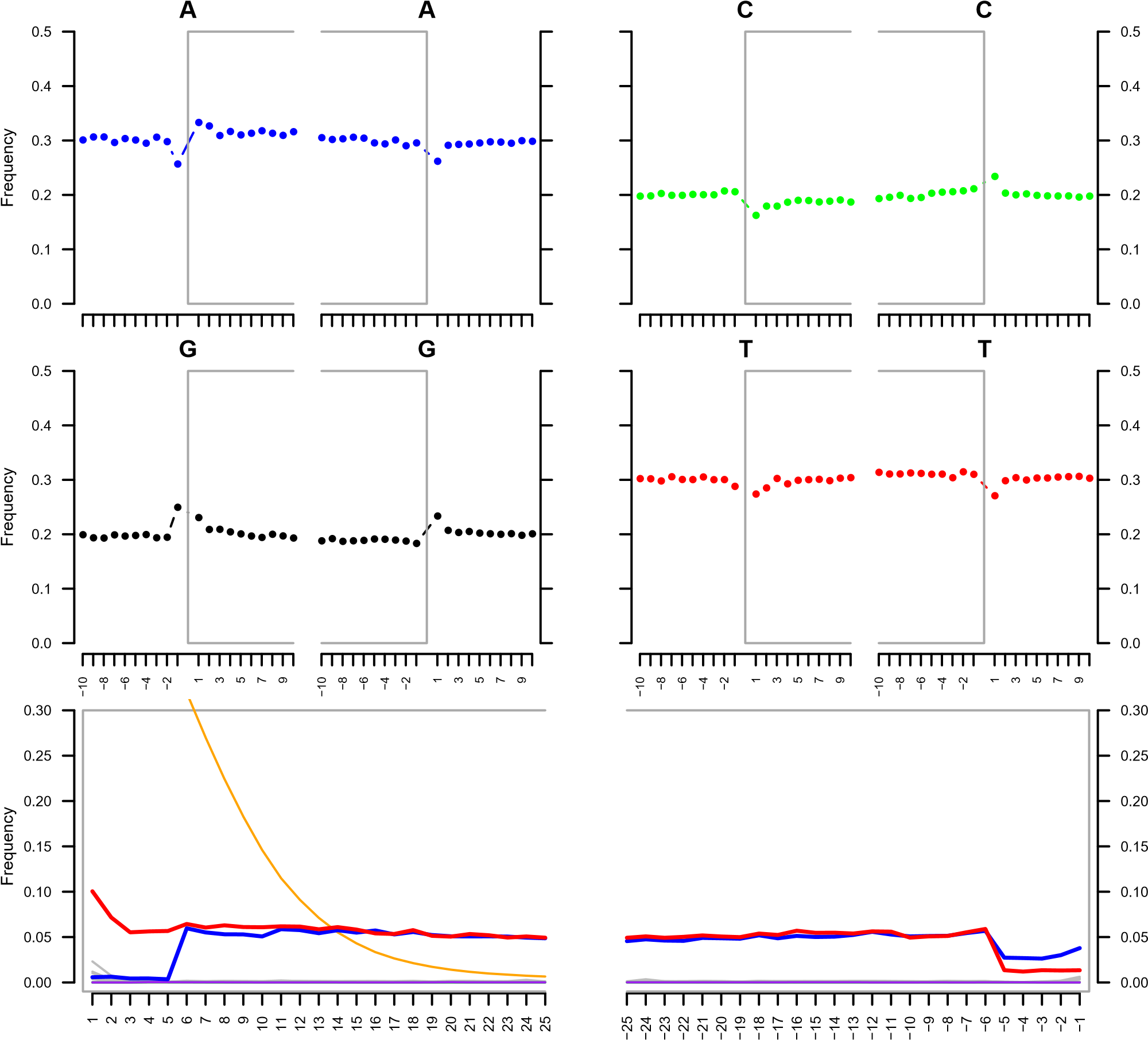


**Fig. S21.** MapDamage fragment misincorporation plot for the Mexico Bellas Artes 23 sample

R0121_MXBA24_align


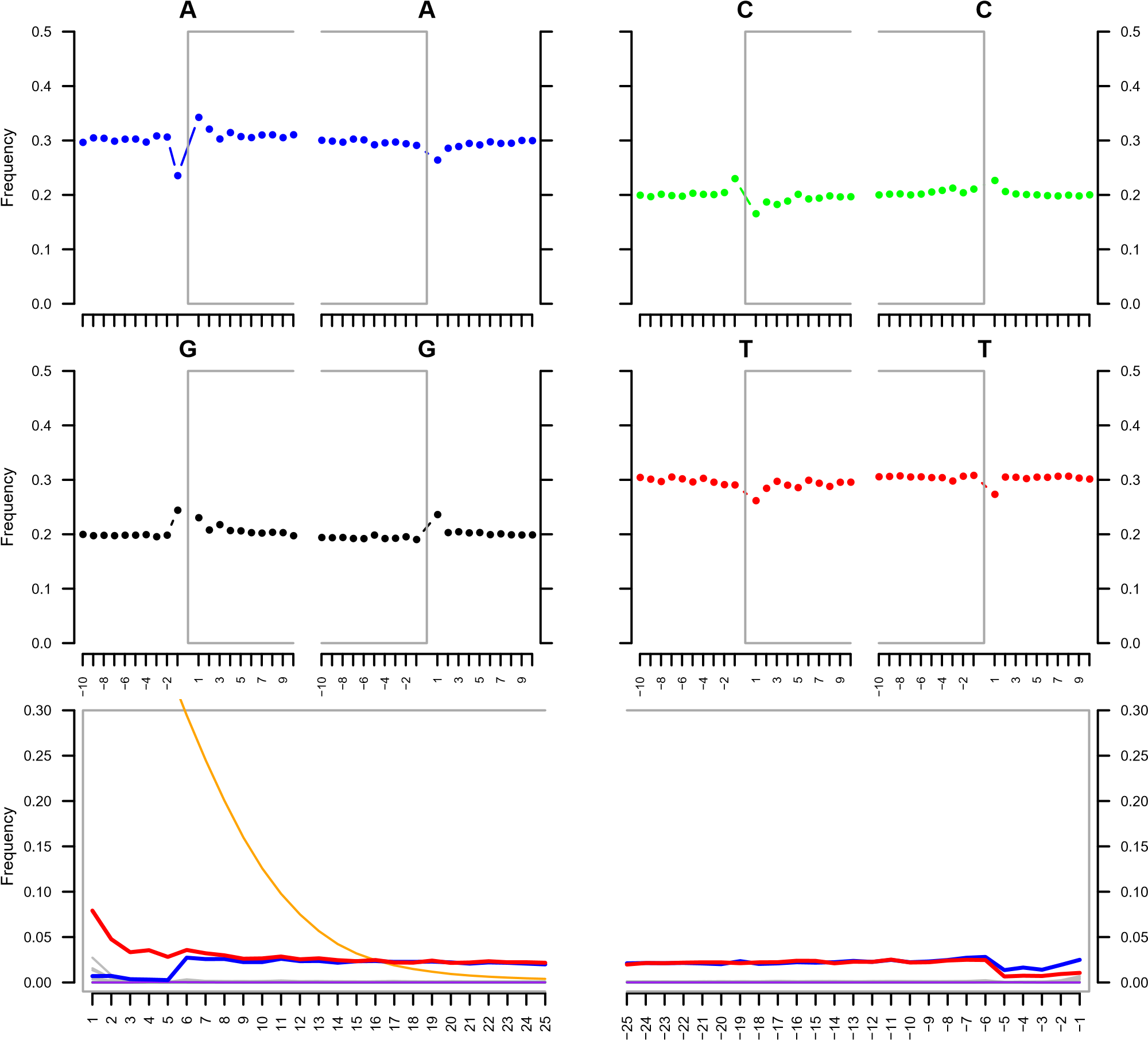


**Fig. S22.** MapDamage fragment misincorporation plot for the Mexico Bellas Artes 24 sample

Table S1. List of modern cattle mitogenomes used in the study.

| GenBank ref | Haplogroup | Country | Breed/variety |
| --- | --- | --- | --- |
| AB074965 | T3 | Japan | Japanese Black |
| AF492351 | T3 |  | Fleckvieh |
| EU177815 | T3 | Italy | Piedmontese |
| EU177816 | T3 | Italy | Chianina |
| EU177817 | T3 | Italy | Valdostana |
| EU177818 | T3 | Italy | Chianina |
| EU177819 | T3 | Italy | Chianina |
| EU177820 | T3 | Italy | Chianina |
| EU177821 | T3 | Italy | Friesian |
| EU177822 | T3 | Italy | Chianina |
| EU177823 | T3 | Italy | Cinisara |
| EU177825 | T3 | Italy | Chianina |
| EU177826 | T3 | Italy | Friesian |
| EU177827 | T3 | Italy | Friesian |
| EU177828 | T3 | Italy | Chianina |
| EU177829 | T3 | Italy | Maremmana |
| EU177830 | T3 | Italy | Podolica |
| EU177831 | T3 | Italy | Modicana |
| EU177832 | T3 | Italy | Pettiazza |
| EU177833 | T3 | Spain | Betizuak |
| EU177834 | T3 | Spain | Betizuak |
| EU177835 | T3 | Iraq | Iraqi |
| EU177836 | T3 | Iraq | Iraqi |
| EU177837 | T3 | Iraq | Iraqi |
| EU177838 | T3 | Iran | Iranian |
| EU177839 | T3 | Iran | Iranian |
| EU177840 | T123 | Italy | Cabannina |
| EU177841 | T1 | Italy | Chianina |
| EU177842 | T1 | Italy | Cinisara |
| EU177843 | T1 | Italy | Podolica |
| EU177844 | T1 | Italy | Maremmana |
| EU177845 | T1 | Italy | Chianina |
| EU177846 | T1 | Italy | Chianina |
| EU177847 | T1 | Italy | Friesian |
| EU177848 | T1 | Iraq | Iraqi |
| EU177849 | T2 | Greece | Greek |
| EU177850 | T2 | Italy | Cabannina |
| EU177851 | T2 | Italy | Cabannina |
| EU177852 | T2 | Greece | Greek |
| EU177853 | T2 | Italy | Chianina |
| EU177854 | T2 | Italy | Chianina |
| EU177855 | T2 | Italy | Chianina |
| EU177856 | T2 | Iraq | Iraqi |
| EU177857 | T2 | Iran | Iranian |
| EU177858 | T2 | Iran | Iranian |
| EU177859 | T2 | Iran | Iranian |
| EU177860 | T2 | Iran | Iranian |
| EU177861 | T2 | Italy | Rendena |
| EU177862 | T5 | Italy | Valdostana |
| EU177863 | T5 | Italy | Piedmontese |
| EU177864 | T5 | Iraq | Iraqi |
| EU177865 | T5 | Iraq | Iraqi |
| EU177866 | Q | Italy | Cabannina |
| EU177867 | Q | Italy | Cabannina |
| EU177868 | I1 | Iraq | Iraqi |
| EU177869 | I1 | Iraq | Iraqi |
| EU177870 | I2 | Iran | Iranian |
| FJ971080 | Q | Italy | Romagnola |
| FJ971081 | Q | Italy | Chianina |
| FJ971082 | Q1 | Italy | Italian Red Pied |
| FJ971083 | Q1 | Italy | Romagnola |
| FJ971084 | R | Italy | Agerolese |
| FJ971085 | R | Italy | Cinisara |
| FJ971086 | R | Italy | Cinisara |
| FJ971087 | R | Italy | Romagnola |
| FJ971088 | I1 | Mongolia | Unknown |
| HQ184030 | Q2 | Italy | Chianina |
| HQ184031 | Q2 | Italy | Chianina |
| HQ184032 | Q2 | Italy | Chianina |
| HQ184033 | Q2 | Italy | Romagnola |
| HQ184034 | Q1a | Italy | Romagnola |
| HQ184035 | Q1a | Italy | Romagnola |
| HQ184036 | Q1 | Italy | Grey Alpine |
| HQ184037 | Q1 | Italy | Grey Alpine |
| HQ184038 | Q1 | Italy | Cabannina |
| HQ184039 | Q1 | Italy | Chianina |
| HQ184040 | R1 | Italy | Romagnola |
| HQ184041 | R1 | Italy | Romagnola |
| HQ184042 | R1 | Italy | Romagnola |
| HQ184043 | R1 | Italy | Romagnola |
| HQ184044 | R1 | Italy | Romagnola |
| HQ184045 | R2 | Italy | Marchigiana |
| JN817300 | T1c1 | Portugal | Alentejana |
| JN817301 | T1c1 | Portugal | Alentejana |
| JN817306 | T1e | Italy | Calvana |
| JN817307 | T1c1 | Mexico | Creole |
| JN817308 | T1a | Mexico | Chihuahua Creole |
| JN817309 | T1c1a1 | Uruguay | Pampa Chaqueno Creole |
| JN817310 | T1c1a1 | Uruguay | Pampa Chaqueno Creole |
| JN817311 | T1c1a1 | Uruguay | Pampa Chaqueno Creole |
| JN817312 | T1a | Italy | Italian Brown |
| JN817313 | T1a | Italy | Chianina |
| JN817314 | T1a | Italy | Chianina |
| JN817315 | T1a | Italy | Chianina |
| JN817316 | T1a | Italy | Chianina |
| JN817317 | T1a | Italy | Cinisara |
| JN817318 | T1a | Italy | Cinisara |
| JN817319 | T1c | Italy | Cinisara |
| JN817320 | T1b1 | Italy | Cinisara |
| JN817321 | T1d1 | Egypt | Domiaty |
| JN817322 | T1c1a1 | Egypt | Domiaty |
| JN817323 | T1c1 | Egypt | Domiaty |
| JN817324 | T1b1 | Egypt | Domiaty |
| JN817325 | T1c | Egypt | Menofi |
| JN817326 | T1c | Egypt | Menofi |
| JN817327 | T1b | Egypt | Menofi |
| JN817328 | T1c | Egypt | Menofi |
| JN817329 | T1f | Egypt | Menofi |
| JN817331 | T1a | France | Limousin |
| JN817332 | T1a | Italy | Maremmana |
| JN817333 | T1a | Italy | Maremmana |
| JN817334 | T1b1 | Italy | Marchigiana |
| JN817335 | T1a | Italy | Marchigiana |
| JN817336 | T1a | Italy | Marchigiana |
| JN817337 | T1a | Italy | Marchigiana |
| JN817338 | T1a | Italy | Marchigiana |
| JN817339 | T1a | Italy | Marchigiana |
| JN817340 | T1a | Italy | Agerolese |
| JN817341 | T1a | Italy | Agerolese |
| JN817342 | T1a | Italy | Agerolese |
| JN817343 | T1f | Italy | Italian Podolian |
| JN817344 | T1a | Italy | Italian Podolian |
| JN817345 | T1a | Italy | Romagnola |
| JN817346 | T1c1a | Italy | Romagnola |
| JN817347 | T1a | Italy | Romagnola |
| JN817348 | T1b | Ethiopia | Sheko |
| JN817349 | T1b1 | Ethiopia | Sheko |
| JN817350 | T1b1 | Italy | Chianina |
| JN817351 | T1b1 | Italy | Marchigiana |
| JQ967333 | T3 | Italy | Italian Red Pied |
| KP637147 | Q1 | Spain | Pirenaica |
| KT184451 | T3 | Egypt | Menofi |
| KT184452 | T3 | Egypt | Domiaty |
| KT184453 | T3 | Egypt | Menofi |
| KT184454 | T3 | Egypt | Menofi |
| KT184455 | T3 | Egypt | Menofi |
| KT184456 | T2 | Egypt | Domiaty |
| KT184457 | T2 | Egypt | Domiaty |
| KT184458 | T2 | Egypt | Menofi |
| KT184459 | T2 | Egypt | Menofi |
| KT184460 | T2 | Egypt | Domiaty |
| KT184461 | T2 | Egypt | Menofi |
| KT184462 | T1a | Egypt | Domiaty |
| KT184463 | T1a | Egypt | Domiaty |
| KT184464 | T1a | Egypt | Menofi |
| KT184465 | T1a | Egypt | Menofi |
| KT184466 | T1a | Egypt | Menofi |
| KT184467 | T1b1 | Egypt | Menofi |
| KT184468 | T1b1 | Egypt | Menofi |
| KT184469 | T1b1 | Egypt | Domiaty |
| KT184470 | T1c | Egypt | Domiaty |
| KT184471 | Q1 | Egypt | Domiaty |
| KT184472 | Q1 | Egypt | Domiaty |
| KT343748 | T3c | Malta | Maltese |
| KT343749 | T3d | Malta | Maltese |
| MF169209 | T3 | Denmark | drinking horn sample; 15th century |
| NC_006853 | T3 | Denmark | Korean |
| V00654 | T3 |  | BSR |

Table S2. Puerto Real 2 C->T and G->A misincorporations rates.

|  | Position | C.T | G.A |
| --- | --- | --- | --- |
| 1 | -12 | 0.976164 | 0.987505 |
| 2 | -11 | 0.974708 | 0.986354 |
| 3 | -10 | 0.973111 | 0.985126 |
| 4 | -9 | 0.971297 | 0.983858 |
| 5 | -8 | 0.968738 | 0.982858 |
| 6 | -7 | 0.964206 | 0.982665 |
| 7 | -6 | 0.955543 | 0.983424 |
| 8 | -5 | 0.940405 | 0.984388 |
| 9 | -4 | 0.922762 | 0.98454 |
| 10 | -3 | 0.908303 | 0.983963 |
| 11 | -2 | 0.89775 | 0.98297 |
| 12 | -1 | 0.888829 | 0.981767 |
| 13 | 1 | 0.97132 | 0.803241 |
| 14 | 2 | 0.973166 | 0.829147 |
| 15 | 3 | 0.974818 | 0.856144 |
| 16 | 4 | 0.975705 | 0.904008 |
| 17 | 5 | 0.975011 | 0.948339 |
| 18 | 6 | 0.972419 | 0.970462 |
| 19 | 7 | 0.97012 | 0.978747 |
| 20 | 8 | 0.969943 | 0.982102 |
| 21 | 9 | 0.97153 | 0.983714 |
| 22 | 10 | 0.973635 | 0.984805 |
| 23 | 11 | 0.975666 | 0.985774 |
| 24 | 12 | 0.977596 | 0.986644 |

Table S3. Puerto Real 4 C->T and G->A misincorporations rates.

|  | Position | C.T | G.A |
| --- | --- | --- | --- |
| 1 | -12 | 0.966809 | 0.982507 |
| 2 | -11 | 0.96561 | 0.981787 |
| 3 | -10 | 0.96457 | 0.980961 |
| 4 | -9 | 0.963703 | 0.980014 |
| 5 | -8 | 0.962274 | 0.979373 |
| 6 | -7 | 0.958729 | 0.979803 |
| 7 | -6 | 0.950769 | 0.981446 |
| 8 | -5 | 0.935341 | 0.983405 |
| 9 | -4 | 0.916942 | 0.98439 |
| 10 | -3 | 0.903135 | 0.984554 |
| 11 | -2 | 0.892212 | 0.984446 |
| 12 | -1 | 0.879105 | 0.984353 |
| 13 | 1 | 0.974351 | 0.792698 |
| 14 | 2 | 0.974979 | 0.80986 |
| 15 | 3 | 0.975533 | 0.832886 |
| 16 | 4 | 0.975397 | 0.889355 |
| 17 | 5 | 0.973612 | 0.941564 |
| 18 | 6 | 0.969692 | 0.966104 |
| 19 | 7 | 0.966092 | 0.974613 |
| 20 | 8 | 0.964876 | 0.97763 |
| 21 | 9 | 0.965695 | 0.978704 |
| 22 | 10 | 0.967004 | 0.979389 |
| 23 | 11 | 0.967991 | 0.980294 |
| 24 | 12 | 0.968802 | 0.981295 |

Table S4. Puerto Real 5 C->T and G->A misincorporations rates.

|  | Position | C.T | G.A |
| --- | --- | --- | --- |
| 1 | -12 | 0.958445 | 0.985285 |
| 2 | -11 | 0.964118 | 0.981664 |
| 3 | -10 | 0.965562 | 0.978525 |
| 4 | -9 | 0.965696 | 0.975596 |
| 5 | -8 | 0.965313 | 0.972644 |
| 6 | -7 | 0.961162 | 0.97351 |
| 7 | -6 | 0.951676 | 0.976763 |
| 8 | -5 | 0.936151 | 0.979402 |
| 9 | -4 | 0.920512 | 0.980224 |
| 10 | -3 | 0.905451 | 0.980296 |
| 11 | -2 | 0.884684 | 0.980381 |
| 12 | -1 | 0.855871 | 0.98042 |
| 13 | 1 | 0.968652 | 0.672231 |
| 14 | 2 | 0.970051 | 0.707308 |
| 15 | 3 | 0.971219 | 0.764941 |
| 16 | 4 | 0.971624 | 0.856578 |
| 17 | 5 | 0.969809 | 0.933148 |
| 18 | 6 | 0.964528 | 0.965049 |
| 19 | 7 | 0.958119 | 0.975816 |
| 20 | 8 | 0.955728 | 0.979407 |
| 21 | 9 | 0.959655 | 0.979746 |
| 22 | 10 | 0.963911 | 0.979642 |
| 23 | 11 | 0.968322 | 0.978913 |
| 24 | 12 | 0.973736 | 0.975613 |

Table S5. Puerto Real 6 C->T and G->A misincorporations rates.

|  | Position | C.T | G.A |
| --- | --- | --- | --- |
| 1 | -12 | 0.950871 | 0.974366 |
| 2 | -11 | 0.952711 | 0.974662 |
| 3 | -10 | 0.953616 | 0.975368 |
| 4 | -9 | 0.954506 | 0.975994 |
| 5 | -8 | 0.955107 | 0.976695 |
| 6 | -7 | 0.953923 | 0.978155 |
| 7 | -6 | 0.949001 | 0.980592 |
| 8 | -5 | 0.93791 | 0.983315 |
| 9 | -4 | 0.924568 | 0.985083 |
| 10 | -3 | 0.913818 | 0.986072 |
| 11 | -2 | 0.907367 | 0.986647 |
| 12 | -1 | 0.896428 | 0.987273 |
| 13 | 1 | 0.978696 | 0.854013 |
| 14 | 2 | 0.978085 | 0.873036 |
| 15 | 3 | 0.977471 | 0.885504 |
| 16 | 4 | 0.976273 | 0.912788 |
| 17 | 5 | 0.973508 | 0.945365 |
| 18 | 6 | 0.968529 | 0.964439 |
| 19 | 7 | 0.963577 | 0.971192 |
| 20 | 8 | 0.960789 | 0.972752 |
| 21 | 9 | 0.959784 | 0.972304 |
| 22 | 10 | 0.958586 | 0.971926 |
| 23 | 11 | 0.95626 | 0.97235 |
| 24 | 12 | 0.954216 | 0.972337 |

Table S6. Puerto Real 7 C->T and G->A misincorporations rates.

|  | Position | C.T | G.A |
| --- | --- | --- | --- |
| 1 | -12 | 0.970126 | 0.980827 |
| 2 | -11 | 0.96685 | 0.981399 |
| 3 | -10 | 0.964273 | 0.981506 |
| 4 | -9 | 0.962826 | 0.981135 |
| 5 | -8 | 0.960429 | 0.981333 |
| 6 | -7 | 0.957629 | 0.981715 |
| 7 | -6 | 0.954839 | 0.982075 |
| 8 | -5 | 0.952026 | 0.982424 |
| 9 | -4 | 0.950008 | 0.982573 |
| 10 | -3 | 0.947466 | 0.982853 |
| 11 | -2 | 0.934202 | 0.984604 |
| 12 | -1 | 0.891034 | 0.986829 |
| 13 | 1 | 0.977729 | 0.860998 |
| 14 | 2 | 0.977722 | 0.872366 |
| 15 | 3 | 0.977894 | 0.87362 |
| 16 | 4 | 0.977659 | 0.897437 |
| 17 | 5 | 0.976136 | 0.938954 |
| 18 | 6 | 0.972903 | 0.96349 |
| 19 | 7 | 0.969241 | 0.973367 |
| 20 | 8 | 0.966505 | 0.977655 |
| 21 | 9 | 0.965322 | 0.979709 |
| 22 | 10 | 0.966159 | 0.980419 |
| 23 | 11 | 0.967114 | 0.981237 |
| 24 | 12 | 0.967132 | 0.982766 |

Table S7. Puerto Real 8 C->T and G->A misincorporations rates.

|  | Position | C.T | G.A |
| --- | --- | --- | --- |
| 1 | -12 | 0.926897 | 0.969981 |
| 2 | -11 | 0.941122 | 0.967227 |
| 3 | -10 | 0.947489 | 0.963089 |
| 4 | -9 | 0.950953 | 0.959332 |
| 5 | -8 | 0.953303 | 0.955807 |
| 6 | -7 | 0.951486 | 0.958633 |
| 7 | -6 | 0.942708 | 0.966943 |
| 8 | -5 | 0.928562 | 0.9729 |
| 9 | -4 | 0.91817 | 0.975199 |
| 10 | -3 | 0.908432 | 0.976647 |
| 11 | -2 | 0.894278 | 0.978087 |
| 12 | -1 | 0.877514 | 0.979221 |
| 13 | 1 | 0.968327 | 0.675918 |
| 14 | 2 | 0.967718 | 0.763687 |
| 15 | 3 | 0.967009 | 0.818595 |
| 16 | 4 | 0.965517 | 0.875561 |
| 17 | 5 | 0.961127 | 0.930252 |
| 18 | 6 | 0.950763 | 0.959607 |
| 19 | 7 | 0.936489 | 0.970184 |
| 20 | 8 | 0.929989 | 0.972489 |
| 21 | 9 | 0.933898 | 0.97119 |
| 22 | 10 | 0.938952 | 0.96896 |
| 23 | 11 | 0.944749 | 0.964749 |
| 24 | 12 | 0.948731 | 0.954759 |

Table S8. Puerto Real 9 C->T and G->A misincorporations rates.

|  | Position | C.T | G.A |
| --- | --- | --- | --- |
| 1 | -12 | 0.954195 | 0.975926 |
| 2 | -11 | 0.952828 | 0.974142 |
| 3 | -10 | 0.951798 | 0.972162 |
| 4 | -9 | 0.951109 | 0.969945 |
| 5 | -8 | 0.94974 | 0.968251 |
| 6 | -7 | 0.945601 | 0.968636 |
| 7 | -6 | 0.934287 | 0.972015 |
| 8 | -5 | 0.911681 | 0.975752 |
| 9 | -4 | 0.884468 | 0.977584 |
| 10 | -3 | 0.864616 | 0.978018 |
| 11 | -2 | 0.851815 | 0.977941 |
| 12 | -1 | 0.841753 | 0.977744 |
| 13 | 1 | 0.963709 | 0.754884 |
| 14 | 2 | 0.964065 | 0.790922 |
| 15 | 3 | 0.964345 | 0.825991 |
| 16 | 4 | 0.963751 | 0.877919 |
| 17 | 5 | 0.960743 | 0.928667 |
| 18 | 6 | 0.95422 | 0.956497 |
| 19 | 7 | 0.947984 | 0.966957 |
| 20 | 8 | 0.946703 | 0.970307 |
| 21 | 9 | 0.949064 | 0.971308 |
| 22 | 10 | 0.952328 | 0.971813 |
| 23 | 11 | 0.955306 | 0.972536 |
| 24 | 12 | 0.958148 | 0.973384 |

Table S9. Merida 11 C->T and G->A misincorporations rates.

|  | Position | C.T | G.A |
| --- | --- | --- | --- |
| 1 | -12 | 0.962001 | 0.97836 |
| 2 | -11 | 0.960373 | 0.977261 |
| 3 | -10 | 0.958762 | 0.97606 |
| 4 | -9 | 0.956887 | 0.974937 |
| 5 | -8 | 0.954197 | 0.974236 |
| 6 | -7 | 0.949324 | 0.974573 |
| 7 | -6 | 0.93957 | 0.97625 |
| 8 | -5 | 0.921464 | 0.978382 |
| 9 | -4 | 0.901207 | 0.979275 |
| 10 | -3 | 0.887095 | 0.979083 |
| 11 | -2 | 0.876492 | 0.97851 |
| 12 | -1 | 0.86488 | 0.97791 |
| 13 | 1 | 0.964501 | 0.812124 |
| 14 | 2 | 0.965823 | 0.828413 |
| 15 | 3 | 0.966972 | 0.848651 |
| 16 | 4 | 0.967229 | 0.889232 |
| 17 | 5 | 0.9654 | 0.934029 |
| 18 | 6 | 0.960959 | 0.95946 |
| 19 | 7 | 0.957013 | 0.969268 |
| 20 | 8 | 0.955776 | 0.973201 |
| 21 | 9 | 0.956701 | 0.975057 |
| 22 | 10 | 0.95822 | 0.976402 |
| 23 | 11 | 0.959706 | 0.977668 |
| 24 | 12 | 0.9611 | 0.978895 |

Table S10. Merida 12 C->T and G->A misincorporations rates.

|  | Position | C.T | G.A |
| --- | --- | --- | --- |
| 1 | -12 | 0.976366 | 0.987912 |
| 2 | -11 | 0.974874 | 0.986738 |
| 3 | -10 | 0.973036 | 0.985586 |
| 4 | -9 | 0.970989 | 0.984378 |
| 5 | -8 | 0.968353 | 0.983313 |
| 6 | -7 | 0.963823 | 0.98295 |
| 7 | -6 | 0.954442 | 0.983709 |
| 8 | -5 | 0.935418 | 0.9849 |
| 9 | -4 | 0.910304 | 0.985194 |
| 10 | -3 | 0.886997 | 0.984726 |
| 11 | -2 | 0.870587 | 0.983774 |
| 12 | -1 | 0.858661 | 0.982558 |
| 13 | 1 | 0.971022 | 0.780979 |
| 14 | 2 | 0.973123 | 0.797062 |
| 15 | 3 | 0.974924 | 0.827313 |
| 16 | 4 | 0.975861 | 0.892549 |
| 17 | 5 | 0.975154 | 0.946218 |
| 18 | 6 | 0.972601 | 0.970062 |
| 19 | 7 | 0.970679 | 0.978364 |
| 20 | 8 | 0.970886 | 0.981698 |
| 21 | 9 | 0.972591 | 0.983372 |
| 22 | 10 | 0.974524 | 0.984665 |
| 23 | 11 | 0.976268 | 0.985896 |
| 24 | 12 | 0.978087 | 0.9869 |

Table S11. Merida 13 C->T and G->A misincorporations rates.

|  | Position | C.T | G.A |
| --- | --- | --- | --- |
| 1 | -12 | 0.972951 | 0.985794 |
| 2 | -11 | 0.971468 | 0.984962 |
| 3 | -10 | 0.970407 | 0.983803 |
| 4 | -9 | 0.969957 | 0.982121 |
| 5 | -8 | 0.968807 | 0.980725 |
| 6 | -7 | 0.964492 | 0.981244 |
| 7 | -6 | 0.953922 | 0.983338 |
| 8 | -5 | 0.930568 | 0.985433 |
| 9 | -4 | 0.895478 | 0.98627 |
| 10 | -3 | 0.86379 | 0.986165 |
| 11 | -2 | 0.849986 | 0.98551 |
| 12 | -1 | 0.850706 | 0.984528 |
| 13 | 1 | 0.973746 | 0.764673 |
| 14 | 2 | 0.975239 | 0.75901 |
| 15 | 3 | 0.976414 | 0.793718 |
| 16 | 4 | 0.976683 | 0.885607 |
| 17 | 5 | 0.975291 | 0.94596 |
| 18 | 6 | 0.971714 | 0.970378 |
| 19 | 7 | 0.96824 | 0.978689 |
| 20 | 8 | 0.967387 | 0.981673 |
| 21 | 9 | 0.969437 | 0.982462 |
| 22 | 10 | 0.971926 | 0.982825 |
| 23 | 11 | 0.973558 | 0.983652 |
| 24 | 12 | 0.974806 | 0.984653 |

Table S12. Merida 14 C->T and G->A misincorporations rates.

|  | Position | C.T | G.A |
| --- | --- | --- | --- |
| 1 | -12 | 0.975859 | 0.98638 |
| 2 | -11 | 0.973893 | 0.985512 |
| 3 | -10 | 0.971637 | 0.984664 |
| 4 | -9 | 0.969418 | 0.983638 |
| 5 | -8 | 0.96698 | 0.982573 |
| 6 | -7 | 0.963239 | 0.981984 |
| 7 | -6 | 0.955906 | 0.982439 |
| 8 | -5 | 0.942612 | 0.983413 |
| 9 | -4 | 0.927179 | 0.983634 |
| 10 | -3 | 0.91511 | 0.983051 |
| 11 | -2 | 0.906945 | 0.981994 |
| 12 | -1 | 0.900516 | 0.980695 |
| 13 | 1 | 0.971048 | 0.78713 |
| 14 | 2 | 0.973164 | 0.779398 |
| 15 | 3 | 0.974859 | 0.805859 |
| 16 | 4 | 0.975593 | 0.88516 |
| 17 | 5 | 0.974554 | 0.945103 |
| 18 | 6 | 0.971486 | 0.969796 |
| 19 | 7 | 0.969154 | 0.978008 |
| 20 | 8 | 0.969433 | 0.981027 |
| 21 | 9 | 0.971067 | 0.98262 |
| 22 | 10 | 0.97272 | 0.984004 |
| 23 | 11 | 0.973897 | 0.98551 |
| 24 | 12 | 0.974919 | 0.986912 |

Table S13. Merida 15 C->T and G->A misincorporations rates.

|  | Position | C.T | G.A |
| --- | --- | --- | --- |
| 1 | -12 | 0.976166 | 0.987163 |
| 2 | -11 | 0.974271 | 0.986061 |
| 3 | -10 | 0.972261 | 0.984847 |
| 4 | -9 | 0.970152 | 0.983497 |
| 5 | -8 | 0.967484 | 0.982265 |
| 6 | -7 | 0.963063 | 0.981722 |
| 7 | -6 | 0.954662 | 0.982232 |
| 8 | -5 | 0.939498 | 0.983184 |
| 9 | -4 | 0.921216 | 0.983326 |
| 10 | -3 | 0.906252 | 0.98262 |
| 11 | -2 | 0.894905 | 0.981451 |
| 12 | -1 | 0.88377 | 0.98011 |
| 13 | 1 | 0.969166 | 0.775083 |
| 14 | 2 | 0.971474 | 0.791135 |
| 15 | 3 | 0.97343 | 0.823483 |
| 16 | 4 | 0.974443 | 0.890478 |
| 17 | 5 | 0.973711 | 0.944734 |
| 18 | 6 | 0.970969 | 0.969186 |
| 19 | 7 | 0.968805 | 0.977839 |
| 20 | 8 | 0.969033 | 0.981283 |
| 21 | 9 | 0.970942 | 0.983005 |
| 22 | 10 | 0.973125 | 0.984317 |
| 23 | 11 | 0.975013 | 0.985616 |
| 24 | 12 | 0.976722 | 0.986838 |

Table S14. Merida 16 C->T and G->A misincorporations rates.

|  | Position | C.T | G.A |
| --- | --- | --- | --- |
| 1 | -12 | 0.977467 | 0.98791 |
| 2 | -11 | 0.975715 | 0.986978 |
| 3 | -10 | 0.973836 | 0.985971 |
| 4 | -9 | 0.971919 | 0.984831 |
| 5 | -8 | 0.969459 | 0.983824 |
| 6 | -7 | 0.965158 | 0.98352 |
| 7 | -6 | 0.956689 | 0.98418 |
| 8 | -5 | 0.940489 | 0.985216 |
| 9 | -4 | 0.91996 | 0.985472 |
| 10 | -3 | 0.902839 | 0.984959 |
| 11 | -2 | 0.890776 | 0.984019 |
| 12 | -1 | 0.881122 | 0.982863 |
| 13 | 1 | 0.972614 | 0.767512 |
| 14 | 2 | 0.974518 | 0.787478 |
| 15 | 3 | 0.976151 | 0.822482 |
| 16 | 4 | 0.976962 | 0.893336 |
| 17 | 5 | 0.976217 | 0.947611 |
| 18 | 6 | 0.973668 | 0.971078 |
| 19 | 7 | 0.971605 | 0.979229 |
| 20 | 8 | 0.971589 | 0.982487 |
| 21 | 9 | 0.973146 | 0.984075 |
| 22 | 10 | 0.975012 | 0.985256 |
| 23 | 11 | 0.97662 | 0.986437 |
| 24 | 12 | 0.978088 | 0.987547 |

Table S15. Mexico Xochimilco 17 C->T and G->A misincorporations rates.

|  | Position | C.T | G.A |
| --- | --- | --- | --- |
| 1 | -12 | 0.7845 | 0.87434 |
| 2 | -11 | 0.778597 | 0.870757 |
| 3 | -10 | 0.77523 | 0.867855 |
| 4 | -9 | 0.773805 | 0.865318 |
| 5 | -8 | 0.772822 | 0.863633 |
| 6 | -7 | 0.769759 | 0.864161 |
| 7 | -6 | 0.761247 | 0.868496 |
| 8 | -5 | 0.747252 | 0.875224 |
| 9 | -4 | 0.735949 | 0.879757 |
| 10 | -3 | 0.73304 | 0.880674 |
| 11 | -2 | 0.739147 | 0.878072 |
| 12 | -1 | 0.750387 | 0.87282 |
| 13 | 1 | 0.826697 | 0.776276 |
| 14 | 2 | 0.826643 | 0.77678 |
| 15 | 3 | 0.826997 | 0.776151 |
| 16 | 4 | 0.824975 | 0.783139 |
| 17 | 5 | 0.813756 | 0.81121 |
| 18 | 6 | 0.79145 | 0.844663 |
| 19 | 7 | 0.772355 | 0.862379 |
| 20 | 8 | 0.764995 | 0.868683 |
| 21 | 9 | 0.766327 | 0.87008 |
| 22 | 10 | 0.771126 | 0.870481 |
| 23 | 11 | 0.776821 | 0.871902 |
| 24 | 12 | 0.784083 | 0.874611 |

Table S16. Mexico Xochimilco 18 C->T and G->A misincorporations rates.

|  | Position | C.T | G.A |
| --- | --- | --- | --- |
| 1 | -12 | 0.959989 | 0.978843 |
| 2 | -11 | 0.958012 | 0.977796 |
| 3 | -10 | 0.955982 | 0.976676 |
| 4 | -9 | 0.95391 | 0.975474 |
| 5 | -8 | 0.951404 | 0.974396 |
| 6 | -7 | 0.947545 | 0.973883 |
| 7 | -6 | 0.941047 | 0.974246 |
| 8 | -5 | 0.931194 | 0.975106 |
| 9 | -4 | 0.921367 | 0.975305 |
| 10 | -3 | 0.91412 | 0.974712 |
| 11 | -2 | 0.908072 | 0.973788 |
| 12 | -1 | 0.901552 | 0.972831 |
| 13 | 1 | 0.961463 | 0.844618 |
| 14 | 2 | 0.963106 | 0.855949 |
| 15 | 3 | 0.964656 | 0.867072 |
| 16 | 4 | 0.965257 | 0.895799 |
| 17 | 5 | 0.963661 | 0.934193 |
| 18 | 6 | 0.959399 | 0.958476 |
| 19 | 7 | 0.955741 | 0.968253 |
| 20 | 8 | 0.954992 | 0.972146 |
| 21 | 9 | 0.956404 | 0.973949 |
| 22 | 10 | 0.958301 | 0.975275 |
| 23 | 11 | 0.960015 | 0.976604 |
| 24 | 12 | 0.961671 | 0.977855 |

Table S17. Mexico Justo Sierra 19 C->T and G->A misincorporations rates.

|  | Position | C.T | G.A |
| --- | --- | --- | --- |
| 1 | -12 | 0.833209 | 0.903148 |
| 2 | -11 | 0.827444 | 0.89672 |
| 3 | -10 | 0.819602 | 0.892179 |
| 4 | -9 | 0.812587 | 0.88763 |
| 5 | -8 | 0.806198 | 0.883249 |
| 6 | -7 | 0.795751 | 0.882205 |
| 7 | -6 | 0.775358 | 0.886848 |
| 8 | -5 | 0.741857 | 0.894836 |
| 9 | -4 | 0.708007 | 0.899907 |
| 10 | -3 | 0.689718 | 0.90047 |
| 11 | -2 | 0.696373 | 0.896083 |
| 12 | -1 | 0.715026 | 0.888367 |
| 13 | 1 | 0.840162 | 0.727814 |
| 14 | 2 | 0.844226 | 0.723035 |
| 15 | 3 | 0.846956 | 0.728158 |
| 16 | 4 | 0.844562 | 0.762125 |
| 17 | 5 | 0.834578 | 0.812479 |
| 18 | 6 | 0.817382 | 0.854385 |
| 19 | 7 | 0.805673 | 0.875133 |
| 20 | 8 | 0.805075 | 0.884023 |
| 21 | 9 | 0.810753 | 0.888872 |
| 22 | 10 | 0.81822 | 0.893113 |
| 23 | 11 | 0.824572 | 0.89863 |
| 24 | 12 | 0.832427 | 0.903651 |

Table S18. Mexico Justo Sierra 20 C->T and G->A misincorporations rates.

|  | Position | C.T | G.A |
| --- | --- | --- | --- |
| 1 | -12 | 0.82421 | 0.890211 |
| 2 | -11 | 0.815372 | 0.88992 |
| 3 | -10 | 0.808407 | 0.889367 |
| 4 | -9 | 0.804703 | 0.887651 |
| 5 | -8 | 0.803426 | 0.885197 |
| 6 | -7 | 0.80032 | 0.884604 |
| 7 | -6 | 0.791425 | 0.887945 |
| 8 | -5 | 0.775269 | 0.894393 |
| 9 | -4 | 0.758356 | 0.899766 |
| 10 | -3 | 0.747769 | 0.902343 |
| 11 | -2 | 0.749654 | 0.901261 |
| 12 | -1 | 0.760987 | 0.897129 |
| 13 | 1 | 0.864313 | 0.721653 |
| 14 | 2 | 0.864034 | 0.727097 |
| 15 | 3 | 0.863003 | 0.738603 |
| 16 | 4 | 0.858294 | 0.770799 |
| 17 | 5 | 0.846143 | 0.819051 |
| 18 | 6 | 0.825685 | 0.859386 |
| 19 | 7 | 0.809251 | 0.878269 |
| 20 | 8 | 0.803921 | 0.884871 |
| 21 | 9 | 0.806603 | 0.886417 |
| 22 | 10 | 0.810668 | 0.88788 |
| 23 | 11 | 0.813645 | 0.891077 |
| 24 | 12 | 0.816614 | 0.895415 |

Table S19. Mexico Justo Sierra 21 C->T and G->A misincorporations rates.

|  | Position | C.T | G.A |
| --- | --- | --- | --- |
| 1 | -12 | 0.826849 | 0.909151 |
| 2 | -11 | 0.834455 | 0.914358 |
| 3 | -10 | 0.84225 | 0.918022 |
| 4 | -9 | 0.847246 | 0.921985 |
| 5 | -8 | 0.846978 | 0.927464 |
| 6 | -7 | 0.843341 | 0.933142 |
| 7 | -6 | 0.839086 | 0.937883 |
| 8 | -5 | 0.837486 | 0.94109 |
| 9 | -4 | 0.840461 | 0.942725 |
| 10 | -3 | 0.84755 | 0.943034 |
| 11 | -2 | 0.859827 | 0.941378 |
| 12 | -1 | 0.876699 | 0.936496 |
| 13 | 1 | 0.905951 | 0.908228 |
| 14 | 2 | 0.906014 | 0.903074 |
| 15 | 3 | 0.905866 | 0.897336 |
| 16 | 4 | 0.904537 | 0.893272 |
| 17 | 5 | 0.901114 | 0.893182 |
| 18 | 6 | 0.894796 | 0.897651 |
| 19 | 7 | 0.886124 | 0.903341 |
| 20 | 8 | 0.876437 | 0.907324 |
| 21 | 9 | 0.86788 | 0.907852 |
| 22 | 10 | 0.860729 | 0.905492 |
| 23 | 11 | 0.853707 | 0.901458 |
| 24 | 12 | 0.845232 | 0.896782 |

Table S20. Mexico Bellas Artes 22 C->T and G->A misincorporations rates.

|  | Position | C.T | G.A |
| --- | --- | --- | --- |
| 1 | -12 | 0.908276 | 0.950153 |
| 2 | -11 | 0.904163 | 0.947613 |
| 3 | -10 | 0.900433 | 0.945139 |
| 4 | -9 | 0.897025 | 0.942787 |
| 5 | -8 | 0.893204 | 0.941007 |
| 6 | -7 | 0.887454 | 0.940623 |
| 7 | -6 | 0.877564 | 0.942314 |
| 8 | -5 | 0.862946 | 0.945198 |
| 9 | -4 | 0.849426 | 0.94695 |
| 10 | -3 | 0.840937 | 0.947273 |
| 11 | -2 | 0.836525 | 0.946821 |
| 12 | -1 | 0.834321 | 0.946066 |
| 13 | 1 | 0.924364 | 0.788169 |
| 14 | 2 | 0.924526 | 0.803386 |
| 15 | 3 | 0.924868 | 0.81677 |
| 16 | 4 | 0.923823 | 0.843132 |
| 17 | 5 | 0.918705 | 0.884018 |
| 18 | 6 | 0.908479 | 0.916868 |
| 19 | 7 | 0.899768 | 0.932167 |
| 20 | 8 | 0.897179 | 0.93848 |
| 21 | 9 | 0.899397 | 0.941309 |
| 22 | 10 | 0.903341 | 0.943332 |
| 23 | 11 | 0.907349 | 0.945648 |
| 24 | 12 | 0.911467 | 0.948202 |

Table S21. Mexico Bellas Artes 23 C->T and G->A misincorporations rates.

|  | Position | C.T | G.A |
| --- | --- | --- | --- |
| 1 | -12 | 0.976929 | 0.987628 |
| 2 | -11 | 0.975306 | 0.986724 |
| 3 | -10 | 0.973516 | 0.985786 |
| 4 | -9 | 0.971671 | 0.984743 |
| 5 | -8 | 0.969545 | 0.983711 |
| 6 | -7 | 0.966406 | 0.983054 |
| 7 | -6 | 0.96091 | 0.983148 |
| 8 | -5 | 0.951938 | 0.983696 |
| 9 | -4 | 0.942055 | 0.983728 |
| 10 | -3 | 0.933411 | 0.983198 |
| 11 | -2 | 0.923931 | 0.982547 |
| 12 | -1 | 0.911808 | 0.981932 |
| 13 | 1 | 0.973431 | 0.789911 |
| 14 | 2 | 0.975087 | 0.813937 |
| 15 | 3 | 0.976473 | 0.849579 |
| 16 | 4 | 0.977057 | 0.907517 |
| 17 | 5 | 0.97607 | 0.952325 |
| 18 | 6 | 0.973167 | 0.972873 |
| 19 | 7 | 0.97088 | 0.980085 |
| 20 | 8 | 0.970968 | 0.982847 |
| 21 | 9 | 0.972575 | 0.984203 |
| 22 | 10 | 0.974437 | 0.985236 |
| 23 | 11 | 0.976008 | 0.98631 |
| 24 | 12 | 0.977428 | 0.987339 |

Table S22. Mexico Bellas Artes 24 C->T and G->A misincorporations rates.

|  | Position | C.T | G.A |
| --- | --- | --- | --- |
| 1 | -12 | 0.946297 | 0.971138 |
| 2 | -11 | 0.944587 | 0.970197 |
| 3 | -10 | 0.942896 | 0.969384 |
| 4 | -9 | 0.941138 | 0.968745 |
| 5 | -8 | 0.938816 | 0.968528 |
| 6 | -7 | 0.93474 | 0.969197 |
| 7 | -6 | 0.927482 | 0.970847 |
| 8 | -5 | 0.916748 | 0.97279 |
| 9 | -4 | 0.907439 | 0.973842 |
| 10 | -3 | 0.902359 | 0.974074 |
| 11 | -2 | 0.898741 | 0.974114 |
| 12 | -1 | 0.894534 | 0.974245 |
| 13 | 1 | 0.962522 | 0.825828 |
| 14 | 2 | 0.962792 | 0.831409 |
| 15 | 3 | 0.963095 | 0.837444 |
| 16 | 4 | 0.962452 | 0.871517 |
| 17 | 5 | 0.959421 | 0.91971 |
| 18 | 6 | 0.953276 | 0.949135 |
| 19 | 7 | 0.94726 | 0.960733 |
| 20 | 8 | 0.944243 | 0.965175 |
| 21 | 9 | 0.944198 | 0.966891 |
| 22 | 10 | 0.945439 | 0.967839 |
| 23 | 11 | 0.946813 | 0.968849 |
| 24 | 12 | 0.94824 | 0.969973 |
